# Global Comprehensive Literature Review and Meta-Analysis of Brucella spp. in Swine Based on Publications From 2000 to 2020

**DOI:** 10.3389/fvets.2021.630960

**Published:** 2021-05-07

**Authors:** Qing-Long Gong, Yu-Han Sun, Yang Yang, Bo Zhao, Qi Wang, Jian-Ming Li, Gui-Yang Ge, Zi-Yang Chen, Kun Shi, Xue Leng, Ying Zong, Rui Du

**Affiliations:** ^1^College of Chinese Medicine Materials, Jilin Agricultural University, Changchun, China; ^2^College of Animal Science and Technology, Jilin Agricultural University, Changchun, China; ^3^Laboratory of Production and Product Application of Sika Deer of Jilin Province, Jilin Agricultural University, Changchun, China; ^4^Key Lab of Animal Production, Product Quality and Security, Ministry of Education, Jilin Agricultural University, Changchun, China

**Keywords:** brucellosis, brucella suis, meta-analysis, prevalence, swine

## Abstract

**Background:** Brucellosis, a zoonotic disease, infects various hosts, including swine and humans. It has reemerged in recent years as a public health concern, and current studies on brucellosis infection in swine have been conducted worldwide. However, no meta-analyses of global brucellosis infection in swine have been published. The aim of this study was to provide an overview of *Brucella* species (spp.) in swine worldwide and the factors associated with its persistence.

**Results:** We searched seven databases for published epidemiological studies on brucellosis in pigs, including the Chinese National Knowledge Infrastructure, Wanfang Data, SpringerLink, ScienceDirect, Web of Science, the VIP Chinese Journal Database and PubMed. We selected 119 articles published from January 1, 2000 to January 3, 2020 for inclusion in the meta-analysis and analyzed the data using a random-effects model. Funnel plots and Egger's test showed significant publication bias in the included studies. The results of the sensitivity analysis showed that our study was relatively stable and reliable. The prevalence of brucellosis in swine was 2.1% (95% CI: 1.6–2.6), of which the highest infection rate, which was found in Europe, was 17.4% (95% CI: 11.1–24.9). The prevalence in feral pigs (15.0%, 95% CI: 8.4–23.2) was higher than that in domestic pigs (1.1%, 95% CI 0.2–2.5). The prevalence in high-income countries (15.7%, 95% CI 8.0–25.3) was significantly higher than that in middle- (0.8%, 95% CI 0.5–1.1), and low-income countries (0.1%, 95% CI 0.0–0.2). The prevalence was highest in finishing pigs at 4.9% (95% CI 0.9–11.0), and lowest among suckling pigs at 0% (95% CI 0.0–0.5).

**Conclusion:** The *Brucella* prevalence in pig herds currently is distributed widely throughout the world. In some countries, swine brucellosis may be a neglected zoonotic disease. We recommend long-term monitoring of the prevalence of brucellosis in domestic and wild pig herds. Attention should also be paid to animal welfare on intensive pig farms; controlling the breeding density may play an important role in reducing the spread of brucellosis among pigs.

## Introduction

Brucellosis is a serious zoonotic disease caused by the *Brucella* species (spp), which occurs worldwide, especially in developing countries (1, 2). Although some developed countries have achieved freedom from animal brucellosis, it has reemerged in Japan, Australia and some European countries (Germany, Finland, Austria, Belgium and Italy) during the past 3 years (3–7). Brucellosis has been found in more than 170 countries in six major regions of the world (8). More than 500,000 new human infections are estimated to occur every year and more than 850 million pigs are infected with *Brucella* spp. (9, 10). At present, the prevalence of swine brucellosis varies widely worldwide, with the highest rates in America, North Africa and southern Europe (11, 12). In South America, the positive rate of swine brucellosis antibodies is 9%, and some countries in the European Union have no swine brucellosis while other countries have a positive antibody rate of 22.7%. In China, the positive rate of swine brucellosis antibodies in some areas is 10% (13–15). The prevalence of the disease varies among different regions, but the overall prevalence has been on the rise since the 1990s, which has had a considerable impact on the health of humans and animals and on the economy (16).

In addition to *Brucella suis*, there are 12 *Brucella* spp. currently (*Brucella* ovis, *Brucella* abortus, *Brucella* canis, et al.) and other strains without standing in nomenclature (17). Most of these species mainly infect specific hosts. Although it has been reported that pigs can be infected with different types of *Brucella* besides *Brucella suis* (18), *B. suis* is responsible for brucellosis in pigs. *Brucella suis* is composed of five biovars referred to as 1 through 5 (19, 20). Among them, *Brucella suis* biovars 1, 2 and 3 cause brucellosis in domestic swine, cattle, sheep and even human beings. Although *Brucella suis* is less harmful than *Brucella melitensis* and *Brucella abortus*, brucellosis in pigs caused by it often leads to chronic infection that is not easily detected (21). It may infect the surrounding livestock and other animals, increasing its epidemic scope and widening its range of infection (22). Most human infections derived from swine are caused by *Brucella suis* biovars 1 and 3 (23–27), which easily infect humans through direct exposure, particularly abattoir workers, farmers and veterinarians (28, 29). To date, there is no effective vaccine for *Brucella* (30). More importantly, the antimicrobial resistance of *Brucella* is emerging in brucellosis endemic regions of the world, such as China, Malaysia, Iran, Qatar and Egypt (31). Therefore, we should pay greater attention to its ongoing spread worldwide.

Pigs play a key role globally in providing animal protein in animal husbandry production. Pork is the most consumed land-animal meat, accounting for more than 36% of the world's meat intake, and has maintained a steady growth over the past few decades (32). Brucellosis was once considered to be one of the main diseases affecting the pig industry. In many countries, especially those in the developing world, pig production is usually housed in low biosecurity environments (32). However, as far as we know, systematic analyses of the overall prevalence of brucellosis in pigs worldwide, are scarce. Hence, we conducted a systematic review and meta-analysis of *Brucella* spp. infection worldwide to analyze the pooled prevalence of brucellosis in pigs and to assess potential risk factors associated with brucellosis prevalence.

## Materials and Methods

### Search Strategy

Six databases were used to search the published research literature related to porcine brucellosis, including PubMed, ScienceDirect, SpringerLink, Web of Science, CNKI, Wanfang Data, and the VIP Chinese Journal Database. We retrieved all papers on worldwide *Brucella* spp. infection in swine that were published from January 1, 2000 to January 3, 2020 (the actual sampling dates in those publications were from 1980 to 2019).

In PubMed, the search terms and formulas used were “(“*Brucella suis*” [MeSH] OR *Brucella melitensis biovar suis*) AND (“Swine”[MeSH] OR Suidae OR Pigs OR Warthogs OR Wart Hogs OR Hog, Wart OR Hogs, Wart OR Wart Hog OR Phacochoerus).” In ScienceDirect, we used the terms, “*Brucella suis*,” “swine,” “pig” and “prevalence.” In SpringerLink, we used the terms “*Brucella suis*” and “pigs.” In Web of Science, we used the keywords “*Brucella suis*,” “Swine” and “prevalence” to search for the “TOPIC” (the article topic). We used the term “*Brucella*” (in Chinese) or “*Brucella* spp.” (in Chinese) or “Brucellosis” (in Chinese) in the CNKI database. In Wanfang Data, we used the terms “*Brucella*” (in Chinese) and “pigs” (in Chinese), or “*Brucella* spp.” (in Chinese) and “pigs” (in Chinese), or “Brucellosis” (in Chinese) and “pigs” (in Chinese). The types of articles found in Wanfang Data were limited to “papers in journals, degree theses and conferences.” The search formulas used in the VIP Chinese Journal Database consisted of “Title” or “keywords” = “*Brucella*” (in Chinese) or “Brucellosis” (in Chinese) and “pigs” (in Chinese). The search strategies and search restrictions are reported in Supplementary Material 1. We used different keywords (“*Brucella suis*,” “brucellosis,” “swine,” “pigs,” “prevalence” and “epidemiological investigation”) in each database for search verification; however, no additional qualified studies were found. Endnote (version X9.3.1) was used to catalog the articles retrieved.

Eligible studies were selected in accordance with the following criteria (inclusion criteria):

**Table:** 

• The subjects of the research must be swine.
• The study's aim must be to investigate the prevalence of *Brucella suis* in swine.
• Data must include information on the number of examined pigs and the number of *Brucella suis*-positive pigs.
• The study must be published in Chinese or English.

Articles with the following characteristics were excluded:

**Table:** 

• Articles that did not match the titles and abstracts (see inclusion criteria).
• Repetition of articles or data.
• The hosts were not swine.
• The article was not research study.
• Unable to access the article's full text.
• Published before 2000.
• The article had one or more internal data conflicts.
• The number of samples was <30.

### Data Extraction and Quality Assessments of the Publications

The four reviewers used standardized data collection forms to extract data that were consistent with the criteria to qualify for inclusion in the meta-analysis (33). Any differences between the reviewers or uncertainty about the quality of the research were resolved through the intervention of the lead author (QLG). The following information was reported: first author, the sampling year, the year of publication, income level, geographical region of the study, detection method, age, gender, collection season, feeding mode, pig classification, total number of pig samples and the number of samples that tested positive for *Brucella*.

The quality of the publications was graded using a scoring approach (34). We scored each study, and assigned a score of 5 when the information was described in greater detail (i.e., random sampling, detection method used, sampling method, sampling year and analyses of four or more factors). All the papers were assigned 0-5 points based on the standards. The quality of the papers with 3, 4 or 5 points was considered to be high, papers with a score of 2 points were considered to be average and those with 0 or 1 point were considered to be of low quality.

### Statistical Analysis

Based on a large number of studies, all calculations, including the prevalence of *Brucella* spp. in swine were performed using R software (version 3.5.2). We chose the double-arcsine transformation (PFT) to perform the rate conversions (Table 1), based on these results and those of previous studies (35). The formula of PFT was as followed:

$$\begin{array}{lll} \text{t} & = & \text{arcsin}\left\{\text{sqrt}\left[\text{r}/\left(\text{n}+1\right)\right]\right\}+\text{arcsin}\left\{\text{sqrt}\left[\left(\text{r}+1\right)/\left(\text{n}+\;1\right)\right]\right\}\\ \text{se}\left(\text{t}\right) & = & \text{sqrt}\left[1/\left(\text{n}+\;0.5\right)\right]\\ \text{p} & = & {\left[\text{sin}\left(\text{t}/2\right)\right]}^{2} \end{array}$$

Note: t: transformed prevalence; *r* = positive number; *n* = sample size; se = standard error.

**Table 1:** Normal distribution test of the original rates and the different transformations of the original rates.

**Conversion form**	**W**	**P**
PRAW	0.448	<2.2e−16
PLN	NaN	NA
PLOGIT	NaN	NA
PAS	0.653	2.238e−15
PFT	0.647	1.653e−15

*PRAW, original rate; PLN, logarithmic conversion; PLOGIT, logit transformation; PAS, arcsine transformation; PFT, double-arcsine transformation; NaN, meaningless number; NA, missing data*.

We used forest plots to visualize the results of the analyses and to evaluate the heterogeneity between the studies. Heterogeneity was calculated using Cochran's *Q*-test, the I^2^ statistic and the χ^2^ test (*P* < 0.05), and the cutoff value for the I^2^ statistic was 50%. These two methods were used to examine the degree of statistical significance of the heterogeneity between the selected studies. We used a random effects model for the meta-analysis when heterogeneity was apparent in the selected articles (36). The funnel plot, trim and fill method and Egger's test were used to evaluate the studies for publication bias. Studies have shown that different subgroups may generate different funnel plots because of prevalence changes over time (37). Therefore, a funnel plot and forest plot were used for further evaluation of each subgroup. A sensitivity analysis was conducted to check whether any one study would have a significant impact on the estimates (38).

Heterogeneity between studies is an important indicator in meta-analyses; thus, an accurate assessment of heterogeneity is necessary to finding the key for preventing *Brucella* spp. infection in pigs worldwide. In order to examine the potential sources of heterogeneity, we analyzed the research data using subgroup analyses and univariate regression analysis to identify factors predictive of heterogeneity. The investigated factors consisted of geographical region (comparisons between Asia and other regions), the period of data collection (2006 to 2010 compared to 2000 or before, 2001 to 2005, 2011 to 2015 and 2016 or later), income (comparisons of high- with low- and middle-incomes), detection methods (comparison of the RBPT & TAT with other serological or molecular biology-based methods), season (comparisons of summer with spring, autumn and winter), gender (comparison of boars with sows), pigs' age classifications (comparisons of suckling pigs with finishing, growing and weaning pigs), feeding modes (comparison of extensive farms with intensive farms), pig classification (comparison of feral with domestic pigs) and quality of studies (comparisons of high-quality studies with average-quality studies). This meta-analysis was performed in accordance with the PRISMA guidelines (Supplementary Material 2) (39–41). A correlation analysis was performed for each subgroup by detection method and country in order to track the source of heterogeneity. The heterogeneity of the covariates is represented by R^2^. Our meta-analysis does not include a review agreement and is not registered in the Cochrane database. The code in R for this meta-analysis was presented in Supplementary Material 3.

## Results

### Search Results and Quality of the Eligible Studies

**Figure 1:**
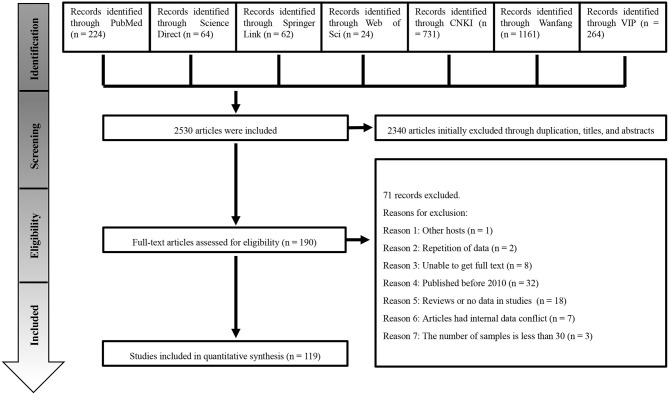
Flow diagram of the search strategies and selection of studies.

A total of 2,530 studies were retrieved from the seven databases. We conducted the meta-analysis with 119 studies based on our inclusion and exclusion criteria (Figure 1). The included articles consisted of 41 high-quality publications (4 or 5 points), 78 average-quality publications (2 or 3 points) and no low-quality publications (0 or 1 point; Supplementary Materials 4, 5).

### Results of Publication Bias

**Figure 2:**
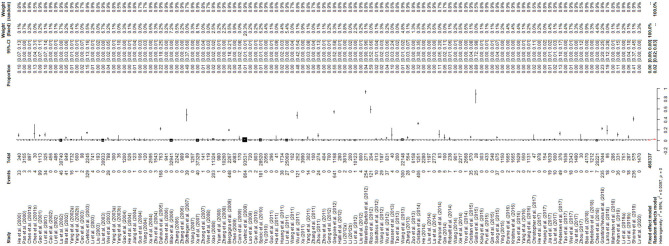
Forest plot of the worldwide prevalence of *Brucella* spp. The length of the horizontal line represents the 95% confidence interval, and the diamonds represent the summarized effect.

**Figure 3:**
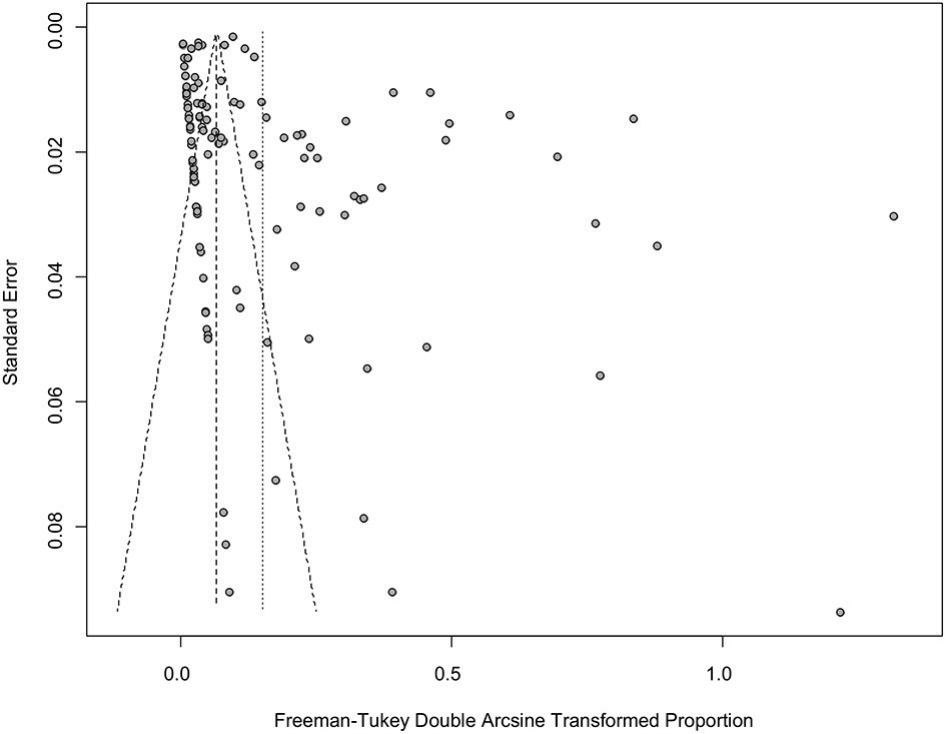
Funnel plot with pseudo 95% confidence intervals for the publication bias test.

The results of the forest plot showed a high degree of heterogeneity between studies (I^2^ = 99.3%, *P* = 0; Figure 2). The funnel plot showed that the graph was asymmetric, indicating the possibility of publication bias or small study effects (Figure 3). Egger's test showed significant publication bias in the included studies (*P* < 0.05; Supplementary Materials 6, 7). The results of the trim and fill method showed that some studies were filled, indicating publication bias or small study effects (Supplementary Material 8). In addition, we evaluated publication bias in all subgroups using funnel plots (Supplementary Materials 9–18).

### Results of Sensitivity Analyses

The results of the sensitivity analysis showed that when a study was omitted, the analysis of the remaining studies yielded the same results as the previous analysis. Therefore, the results of our systematic review and meta-analysis were relatively stable and reliable (Supplementary Material 19).

### Meta-Analysis of *Brucella* spp. in Swine worldwide

Our meta-analysis included five global geographic regions, namely Africa, America, Asia, Europe and Oceania. The pooled prevalence of *Brucella* spp. in swine worldwide was 2.1% (95% CI: 1.6–2.6; Table 2). Among the regional subgroups, the highest prevalence occurred in Europe, which was 17.4% (95% CI: 11.1–24.9; Table 2). Among the countries, Brazil had the highest rate of 93.73% (95% CI 90.5–96.3; Table 2), followed by Spain, with a rate of 59.3% (95% CI: 52.5–66.0; Table 3).

**Table 2:** Pooled worldwide prevalence of *Brucella* spp. by region.

		**No. studies**	**No. tested**	**No. positive**	% (95% CI*)	**Heterogeneity**			**Univariate meta-regression**		Joint analysis*	
						**χ^2^**	***P*-value**	**I^**2**^ (%)**	***P*-value**	**Coefficient (95% CI)**	**R^**2**^-method**	**R^**2**^-country**
Regions*												
	Africa	3	3,661	39	1.7% (0.0–6.7)	111.64	<0.01	98.2%				
	America	9	2,570	382	16.5% (1.3–42.8)	1,580.03	0.00	99.5%				
	Asia	90	320,164	1,429	0.5% (0.3–0.7)	4,029.49	0.00	97.8%	<0.001	−0.295 (−0.3378 to −0.253)	0.00%	68.49%
	Europe	15	133,621	3,575	17.4% (11.1–24.9)	7,805.04	0.00	99.8%				
	Oceania	2	321	16	6.0% (0.5–16.0)	6.73	<0.01	85.1%				
**Sampling years**												
	2000 or before	41	81,246	922	0.7% (0.3–1.4)	2,252.12	0.00	98.2%				
	2001 to 2005	42	140,647	1,182	0.7% (0.4–1.1)	2,182.60	<0.01	98.1%				
	2006 to 2010	35	55,798	1,095	2.7% (1.1–4.8)	4,111.44	0.00	99.2%	<0.001	0.075 (0.039 to 0.112)	12.16%	67.09%
	2011 to 2015	31	32,998	374	0.6% (0.1–1.3)	1,154.60	<0.01	97.4%				
	2016 or later	12	8,965	82	0.7% (0.0–2.0)	289.89	<0.01	96.2%				
Income level*												
	Low	2	3,330	3	0.1% (0.0–0.2)	0.27	–	–				
	Middle	95	445,062	3,093	0.8% (0.5–1.1)	7,877.36	0.00	98.8%				
	High	22	11,945	2,345	15.7% (8.0–25.3)	3,438.12	0.00	99.4%	<0.001	0.297 (0.256 to 0.338)	19.42%	70.65%
Detection method*												
	CFT	6	2,174	295	26.5% (0.0–80.3)	1,682.83	<0.01	99.7%				
	ELISA	11	35,170	1,216	9.4% (2.1–21.0)	3,647.44	0.00	99.7%				
	PCR	3	623	101	16.3% (5.0–32.2)	41.30	<0.01	95.2%				
	RBPT	55	173,023	1,846	1.4% (0.8–2.1)	5,727.35	0.00	99.1%				
	RBPT & TAT	23	105,223	162	0.2% (0.1–0.4)	479.88	<0.01	95.4%	<0.001	−0.152 (−0.206 to −0.103)	0.00%	66.46%
	TAT	17	20,121	275	0.5% (0.0–1.4)	312.29	<0.01	94.9%				
	Others	26	112,102	3,169	11.0% (6.1–17.2)	6,830.99	0.00	99.6%				
Season*												
	Spring	11	3,716	69	2.6% (0.0–3.8)	226.43	<0.01	95.6%				
	Summer	5	1,163	258	11.1% (0.0–63.0)	1,300.31	<0.01	99.7%	0.077	0.195 (−0.021 to 0.411)	36.17%	97.04%
	Autumn	6	1,452	12	0.7% (0.0–3.8)	46.65	<0.01	89.3%				
	Winter	6	3,444	21	1.2% (0.1–3.3)	63.17	<0.01	92.1%				
**Gender**												
	Boars	23	12,737	1282	7.9% (2.4–15.9)	3,594.80	0.00	99.4%	0.314	0.056 (−0.053 to 0.165)	75.00%	82.75%
	Sows	29	51,698	1288	5.1% (2.8–8.1)	3,208.62	0.00	99.1%				
**Age of pigs**												
	Finishing pigs	14	5,778	321	4.9% (0.9–11.0)	684.93	<0.01	98.1%				
	Growing pigs	18	31,454	411	2.1% (0.2–5.2)	1271.98	<0.01	98.7%				
	Suckling pigs	9	1,917	7	0.0% (0.0–0.5)	15.01	0.05	46.7%	0.045	−0.120 (−0.237 to −0.003)	79.25%	87.44%
	Weaning pigs	5	1,015	28	1.0% (0.0–14.2)	98.67	<0.01	95.9%				
**Feeding mode**												
	Extensive	12	34,083	555	2.5% (0.4–5.9)	1,823.79	0.00	99.2%	0.065	0.076 (−0.005 to 0.156)	6.50%	68.57%
	Intensive	35	55,196	468	0.5% (0.1–1.2)	1,790.10	0.00	98.9%				
**Pig classification**												
	Domestic pigs	21	131,196	1,504	1.1% (0.2–2.5)	2,397.46	0.00	99.5%				
	Feral pigs	21	9,186	2,085	15.0% (8.4–23.2)	1,838.57	0.00	99.0%	<0.001	0.277 (0.197 to 0.357)	0.00%	74.40%
**Quality level**												
	Middle	78	203,157	1,201	1.0% (0.6–1.3)	4,344.03	0.00	98.2%				
	High	41	257,180	4,240	5.0% (3.5–6.7)	11,773.05	0.00	99.7%	<0.001	0.117 (0.078 to 0.157)	9.21%	60.97%
Total		119	460,337	5,441	2.1% (1.6–2.6)	16,698.88	0.000	99.3%				

CI^*^, Confidence interval; Joint analysis^*^, Joint analysis with prevalence of detection methods and provinces in China; R^2^, Proportion of between-study variance explained.

Region^*^: Africa: India, Uganda; America: Brazil. USA; Asia: China. India; Europe: Belgium, Croatia, Finland, Germany, Italy, Latvia, Sweden; Oceania: Australia.

Method^*^: CFT: Complement fixation test; ELISA: Enzyme linked immunosorbent assay; PCR: Polymerase chain reaction; RBPT: Rose Bengal plate test; RBPT&TAT: Rose Bengal plate test and Tube agglutination test; TAT: Tube agglutination test.

Season^*^: Spring: Mar. to May.; Summer: Jun. to Aug.; Autumn: Sep. to Nov.; Winter: Dec. to Feb.

*Income level: High: Developed Country; Middle: Developing Country; Low: Least Developed Country*.

**Table 3:** Estimated pooled seroprevalence of *Brucella* spp. by country and region worldwide.

**Countries**	**No. studies**	**Region**	**No. tested**	**No. positive**	**% Prevalence**	**% (95% CI)**
Australia	2	Oceania	321	16	6.0%	0.5–16.0
Belgium	1	Europe	1,168	641	54.9%	52.0–57.7
Brazil	1	America	271	254	93.7%	90.5–96.3
China	89	Asia	319,589	1,193	0.3%	0.2–0.5
Croatia	3	Europe	124,296	1,374	3.5%	1.3–6.8
Egypt	1	Africa	331	36	10.9%	7.7–14.5
Finland	1	Europe	280	0	0.0%	0.0–0.6
Germany	1	Europe	763	168	22.0%	19.2–25.0
India	1	Asia	575	236	41.0%	37.1–45.1
Italy	5	Europe	5,328	915	22.6%	6.7–44.3
Spain	1	Europe	204	121	59.3%	52.5–66.0
Latvia	1	Europe	1,044	235	22.5%	20.0–25.1
Sweden	1	Europe	286	0	0.0%	0.0–0.6
Switzerland	1	Europe	252	121	48.0%	41.9–54.2
Uganda	2	Africa	3,330	3	0.1%	0.0–0.2
USA	8	America	2,299	128	8.7%	2.0–19.2
Total	119		460,337	5,441	2.1%	1.6–2.6

We conducted a subgroup analysis of sampling years, income level, detection method, season, gender, age, feeding mode, pig classification and quality of studies to explore their influence on the prevalence of *Brucella* spp. in swine. Among them, regions, sampling years, income level, detection method, age of pigs, pig classification and quality of the study were identified as risk factors for *Brucella* spp. infection in pigs (*P* < 0.05; Table 2). The combined prevalence of *Brucella* spp. in sampling years 2006 to 2010 was 2.7% (95% CI: 1.1–4.8; Table 2), which was higher than the other four periods. The estimate of prevalence in the high-income group was 15.7% (95% CI: 8.0–25.3; Table 2), which was higher than that of the low- and middle-income groups. In the detection methods subgroup, the CFT showed a prevalence of 26.5% (95% CI: 0.0–80.3; Table 2). The point estimate of the prevalence of *Brucella* spp. in pigs during the summer was the highest at 11.1% (95% CI: 0.0–63.0; Table 2). Compared with the other ages of the pigs, the prevalence of *Brucella* spp. among the finishing pigs (4.9%, 95% CI: 0.9–11.0; Table 2) was higher than that of the growing pigs, suckling pigs and weaning pigs. The prevalence of *Brucella* spp. in feral pigs (15.0%, 95% CI: 8.4–23.2) was significantly higher than that of domestic pigs. The subgroup analysis by quality of study showed the prevalence of *Brucella* spp. in swine was higher in the studies of high quality (5.0%, 95% CI: 3.5–6.7; Table 2). The heterogeneity of each subgroup was explained by detection method (the covariate), which ranged from 0–79.25% (R^2^-method), and countries (the covariate), which was 60.97–97.04% (R^2^-country).

## Discussion

Brucellosis is a zoonotic infectious disease caused by *Brucella*. It is the main cause of infertility, low litter size and miscarriage among sows (42) and an occupational hazard for farmers, slaughterhouse workers and veterinarians (43, 44). The OIE, WHO and Food and Agriculture Organization of the United Nations have classified brucellosis as one of the most important neglected occupational hazards in the world (45, 46). It has had a significant economic impact on the livestock industry and other industries (47). The prevalence of brucellosis plays an important role in the development of the world's pig herds (48). Therefore, we conducted the first meta-analysis to examine the prevalence of brucellosis in pig herds around the world and found that it was unevenly distributed among pigs.

Brucellosis in swine has been widely distributed worldwide for a long time, but in some high-income countries, including Canada, Australia, New Zealand and other countries, the eradication of brucellosis in animal husbandry has been successfully achieved (49, 50). Brucellosis has not been reported in domestic pigs in Belgium since 1969 (51). The United States has implemented reforms in pig management since 1950 to eliminate brucellosis in livestock, and efforts have been made to solve the problem of brucellosis infection in wild animals with almost complete eradication of it in livestock populations (52). However, in our study, the highest prevalence rates were found in America and Europe, and the prevalence rates in the high-income countries were higher than those in middle- and low-income countries. We attribute these results to several factors. First, most of the samples tested in these countries were feral or domestic pigs in contact with wild boars. This was confirmed in the subgroup analysis of pig classification, which showed the prevalence of feral pigs was significantly higher than that of domestic pigs. In recent decades, the population of wild boars has increased rapidly in the United States, which is caused mainly by natural population dynamics, and brucellosis has been reported in wild boars in 14 states (53). In Belgium, the increase in the wild boar population and the prevalence of brucellosis has led to an increased risk of infection in outdoor pig farms (54). Therefore, overabundance of wildlife is considered to be an important factor in the transmission of brucellosis between wildlife and livestock (55). After the first isolation of the *Brucella suis* biovar 2 strain from boars killed by hunters in 1994 (56), *Brucella suis* biovar 2 has been isolated from wild boars in many countries (57–61). Studies have reported that brucellosis among wild boars is widely distributed all over the world (51, 56, 57, 62–65). Second, different modes of feeding in different countries have led to different prevalence rate. Most developed countries mainly focus on intensive farming, while countries with lower incomes focus mainly focus on extensive farming. The prevalence of disease in countries with intensive farming is higher than that in countries with extensive farming, which has been confirmed in several studies (66–68). The increase in herd size results in higher stocking density and worse farm sanitation, thereby promoting the spread of *Brucella* among animals after abortion and parturition (69, 70). We recommend long-term monitoring of wildlife to implement preventive measures before an outbreak of brucellosis. Intensive farms need to control breeding density, pay attention to animal welfare, improve the prevention and control of epidemics and optimize the breeding environment to avoid the large-scale spread of disease. It is worth noting that among the studies we included, only a few on swine brucellosis were conducted in low-income countries. This may indicate that swine brucellosis has been overlooked in these countries and regions. Therefore, although our results show that the prevalence of brucellosis in low-income countries is lower than that of other countries, this finding may be due to these countries' neglect of surveillance and detection of brucellosis. Likewise, the farms in high-income countries are more capable of strengthening their detection of brucellosis, thus, showing a relatively high prevalence. We infer that the actual global infection rate of swine brucellosis may be higher. Although the disease has been controlled or eliminated in some developed countries (such as Canada, New Zealand, Australia and the majority of northern European countries) (71), brucellosis remains an intractable public health problem in poor and underdeveloped countries, especially in the Middle East (72). While strengthening the surveillance and prevention of swine brucellosis in high-incidence areas, we should also continue to strengthen the surveillance in lower incidence areas to prevent widespread infection of swine brucellosis.

The 2006-2010 prevalence of brucellosis was higher than that of the other sampling years. First, as reported in the included studies, an outbreak of swine brucellosis in Jaboticabal, Brazil in 2006 increased the prevalence to 93.7% (73). At the same time, brucellosis was found in Italy after collecting samples from pig farms with breeding problems for serological analysis. Furthermore, detection of suspected cases (non-random sampling) may overestimate the local prevalence of brucellosis (74, 75). Second, during this period, many countries began to analyze the situation of *Brucella* infection in feral pigs. Among the included articles, there were 21 studies on the prevalence of wild boars, and the prevalence of wild boars between 2006 and 2010 was 22.3% (881/3956). There were also studies on isolated *Brucella suis* biovar 2 from wild boars because of the substantial increase in the number of feral pigs (58–60, 76). Moreover, several *Brucella* outbreaks occurred in Germany due to infection of domestic pigs by feral pigs (77). After 2010, the prevalence of brucellosis gradually declined because the OIE proposed controls for *Brucella* farms, giving priority to the development of food safety standards for future animal production (78). Although the prevalence of brucellosis has shown a downward trend, its control should be continued.

The 119 selected studies that were analyzed included five main methods of brucellosis detection: CFT, ELISA, PCR, RBPT and TAT (*P* < 0.001; Table 2). We used detection method as a covariate to perform joint analysis with other risk factors, and the range of heterogeneity explained by the detection method was 0-100.00%, implying that different detection methods had a greater effect on some subgroups.

The analyses of the age and sex of pigs showed that the prevalence of finishing pigs was higher than that of the pigs in the other age groups, and the prevalence of sows was lower than that of the boars. This finding is mainly due to the adult males' contacts with these matrilineal groups during the mating season, while females live in matrilineal groups (60). The prevalence observed in the finishing pigs was higher than that in the other age groups, which was due to the higher involvement of the finishing pigs (79). Although the prevalence of boars was higher than that of sows, no significant differences were found between them, indicating that the relationship between the two animals warrants further examination. We recommend controlling the breeding density; planning a reasonable breeding process may play a positive role in the reduction of the spread of brucellosis in pigs.

The prevalence of brucellosis in the summer was higher than that in the other seasons, but the difference was not significant (Table 2). As far as we know, no research has shown a strong correlation between breeding season and prevalence of swine brucellosis. Studies have shown that the dryness of summer may lead to lack of food and water, increasing the trajectory coverage of animals (80). The correlation analysis showed that countries explained 97.04% of heterogeneity in the seasons subgroup. These results can be interpreted in the context of the world's vast territory with different countries having different characteristics during summer. Meta-analyses showed that the incidence rates of *Brucella* in cattle and deer were higher in hotter and more humid areas (81, 82). Therefore, we speculate that a similar phenomenon occurs in pigs with brucellosis. Thus, efforts to prevent epidemics should be increased in hot and humid areas to create a healthy environment for livestock and reduce the occurrence of disease.

Our study included 41 high-quality articles and 78 average-quality articles. We found that random sampling and detailed descriptions of sampling methods were not included in some of the articles by examining those of average quality. These findings may reflect sampling bias. We recommend that researchers provide detailed descriptions of their sampling and data collection methods to improve the reliability of the data.

This meta-analysis had the advantages of a long-time span, wide coverage and clear methods of analyses, yet it has some limitations. First, the language of the selected articles was limited to English or Chinese, and therefore, qualified articles in other languages might have been overlooked. Second, the articles were obtained from seven databases, which might have excluded qualified articles from other databases. Third, the inadequate information provided by the included studies (e.g., brucellosis classification and geographical factors) might have led to publication bias or other biases in the subgroups (Supplementary Figures 3–12). Fourth, some risk factors were examined in a small number of studies and samples, which might have resulted in small study effects leading to unstable results. We recommend that researchers conduct large-scale studies because the results of small-scale studies are often not representative of the population. Fifth, the research we have included covers only 14 countries, and some of those countries (e.g., Brazil) had few relevant reports. For these countries, we only presented data to reflect global trends, and the results presented are for reference only. The lack of articles from some countries might have led to inaccurate estimates of the prevalence of swine brucellosis in those countries. Prevalence surveys of *Brucella* spp. in more countries are recommended to clarify the true prevalence of swine brucellosis worldwide. Sixth, this study was not registered; however, it was carried out strictly in accordance with the PRISMA guidelines.

In conclusion, the *Brucella* infection rate in pig herds is distributed widely throughout the world. In addition, *Brucella* is common among wild boars in developed countries. Therefore, we suggest carrying out long-term detection of *Brucella* in wild animals and implementing reasonable isolation measures between livestock and wild animals to reduce the chance of contact between them. In addition, countries that do not pay much attention to swine brucellosis should disseminate information about *Brucella* infection, and epidemiological investigations should be conducted as soon as possible to establish better control of the disease. The high prevalence of swine brucellosis will cause serious economic losses to herdsmen, and increase the risk of infection. Therefore, attention to animal welfare on intensive pig farms is crucial, and control of the breeding density may play an important role in reducing the spread of brucellosis in pigs. This study can provide a theoretical basis for researchers to explore control schemes for brucellosis.

## Data Availability Statement

The original contributions presented in the study are included in the article/Supplementary Material, further inquiries can be directed to the corresponding author/s.

## Author Contributions

YZ and RD: idea contributions and funding. BZ, G-YG, Z-YC, and YY: data extraction. Y-HS: database establishment. QW and J-ML: data analysis. Q-LG: writing – original draft. Y-HS, KS, and XL: writing – review and editing. All authors contributed to the article and approved the submitted version.

## Conflict of Interest

The authors declare that the research was conducted in the absence of any commercial or financial relationships that could be construed as a potential conflict of interest.

## Abbreviations

WHO: World Health Organization
LPS: Lipopolysaccharide
S-LPS: Smooth lipopolysaccharide
CNKI: Chinese National Knowledge Infrastructure
PRISMA: Preferred Reporting Items for Systematic Reviews and Meta-Analyses
OIE: Office International Des Epizooties.
