# Supplementary material for: Global Comprehensive Literature Review and Meta-Analysis of Brucella spp. in Swine Based on Publications From 2000 to 2020
Source: Front Vet Sci. 2021 May 7;8:630960. doi: 10.3389/fvets.2021.630960 (PMC8137890; doi:10.3389/fvets.2021.630960)

**Supplementary material 1** Search strategies and restrictions

| Database | Limitation | Search formula* |
| --- | --- | --- |
| PubMed* | All files | ("Brucella suis"[Mesh] OR Brucella melitensis biovar suis) AND ("Swine"[MeSH] OR Suidae OR Pigs OR Warthogs OR Wart Hogs OR Hog, Wart OR Hogs, Wart OR Wart Hog OR Phacochoerus) |
| Springer-Link | All files | “Brucella suis” and “pigs” |
| ScienceDirect | Keywords | “Brucella suis”, “swine”, “pig”, and “prevalence” |
| Web of Science | Keywords & Searched for the TOPIC | “Brucella suis”, “Swine”, and “prevalence” |
| CNKI | Advanced Search & Subject term & Fuzzy retrieval and synonym extension | “Brucella” (Chinese) or “Brucella spp.” (Chinese) or “Brucellosis” (Chinese) |
| Chongqing VIP | Advanced Search & Title or keyword & Fuzzy retrieval and synonym extension | “Brucella” (in Chinese) or “Brucellosis” (in Chinese) and “pigs” (in Chinese).” |
| WanFang | Papers in journals, degree theses, and conferences. Advanced Search & Title or keyword & Fuzzy retrieval and synonym extension | “Brucella” (in Chinese) and “pigs” (in Chinese), or Brucella spp. (in Chinese) and “pigs” (in Chinese), or “Brucellosis” (in Chinese) and “pigs” (in Chinese) |

* The different search strategy was used because different databases have different retrieval logic.

In PubMed, we searched for Medical Subject Headings (MeSH terms; “Brucella suis” and “Swine”) and their entry terms (“Brucella melitensis biovar suis”, “Suidae”, “Pigs”, “Warthogs”, “Wart Hogs”, “Hog, Wart”, “Hogs, Wart”, “Wart Hog”, and “Phacochoerus”) for pigs and swine brucellosis from the “MeSH” option of PubMed. We used the Boolean operator “AND” to connect MeSH terms and “OR” to connect the entry terms. The MeSH terms and their entry terms were respectively combined with “OR” to obtain the search formulas A and B:

| Search formula A: |
| --- |
| ("Brucella suis"[Mesh] OR Brucella melitensis biovar suis) |
| Search formula B: |
| ("Swine"[MeSH] OR Suidae OR Pigs OR Warthogs OR Wart Hogs OR Hog, Wart OR Hogs, Wart OR Wart Hog OR Phacochoerus) |

Then the search formulas A and B were combined with “AND”, so the final search formula was presented as followed:

("Brucella suis"[Mesh] OR Brucella melitensis biovar suis) AND ("Swine"[MeSH] OR Suidae OR Pigs OR Warthogs OR Wart Hogs OR Hog, Wart OR Hogs, Wart OR Wart Hog OR Phacochoerus).

**Supplementary material 2** Items on the PRISMA checklist

| **Section/topic** | **#** | **Checklist item** | **Reported on page #** |
| --- | --- | --- | --- |
| **TITLE** |  |  |  |
| Title | 1 | Global comprehensive literature review and meta-analysis of *Brucella* spp. in swine based on publications from 2000- 2020 | 1 |
| **ABSTRACT** |  |  |  |
| Structured summary | 2 | Provide a structured summary including, as applicable: background; objectives; data sources; study eligibility criteria, participants, and interventions; study appraisal and synthesis methods; results; limitations; conclusions and implications of key findings; systematic review registration number. | 1-2 |
| **INTRODUCTION** |  |  |  |
| Rationale | 3 | Describe the rationale for the review in the context of what is already known. | 3 |
| Objectives | 4 | Provide an explicit statement of questions being addressed with reference to participants, interventions, comparisons, outcomes, and study design (PICOS). | 3 |
| **METHODS** |  |  |  |
| Protocol and registration | 5 | Indicate if a review protocol exists, if and where it can be accessed (e.g., Web address), and, if available, provide registration information including registration number. | 4-5 |
| Eligibility criteria | 6 | Specify study characteristics (e.g., PICOS, length of follow-up) and report characteristics (e.g., years considered, language, publication status) used as criteria for eligibility, giving rationale. | 4-5 |
| Information sources | 7 | Describe all information sources (e.g., databases with dates of coverage, contact with study authors to identify additional studies) in the search and date last searched. | 4-5 |
| Search | 8 | Present full electronic search strategy for at least one database, including any limits used, such that it could be repeated. | 4-5 |
| Study selection | 9 | State the process for selecting studies (i.e., screening, eligibility, included in systematic review, and, if applicable, included in the meta-analysis). | 4-5 |
| Data collection process | 10 | Describe method of data extraction from reports (e.g., piloted forms, independently, in duplicate) and any processes for obtaining and confirming data from investigators. | 4-5 |
| Data items | 11 | List and define all variables for which data were sought (e.g., PICOS, funding sources) and any assumptions and simplifications made. | 4-5 |
| Risk of bias in individual studies | 12 | Describe methods used for assessing risk of bias of individual studies (including specification of whether this was done at the study or outcome level), and how this information is to be used in any data synthesis. | 4-5 |
| Summary measures | 13 | State the principal summary measures (e.g., risk ratio, difference in means). | 4-5 |
| Synthesis of results | 14 | Describe the methods of handling data and combining results of studies, if done, including measures of consistency (e.g., I^2^) for each meta-analysis. | 4-5 |
| Risk of bias across studies | 15 | Specify any assessment of risk of bias that may affect the cumulative evidence (e.g., publication bias, selective reporting within studies). | 4-5 |
| Additional analyses | 16 | Describe methods of additional analyses (e.g., sensitivity or subgroup analyses, meta-regression), if done, indicating which were pre-specified. | 4-5 |
| **RESULTS** |  |  |  |
| Study selection | 17 | Give numbers of studies screened, assessed for eligibility, and included in the review, with reasons for exclusions at each stage, ideally with a flow diagram. | 5-6, Figure 1 |
| Study characteristics | 18 | For each study, present characteristics for which data were extracted (e.g., study size, PICOS, follow-up period) and provide the citations. | 5-6, Tables S2 and S3 |
| Risk of bias within studies | 19 | Present data on risk of bias of each study and, if available, any outcome level assessment (see item 12). | 5-6, Figures 2 and 3, Figures S1-S12 and Tables S2 and S3 |
| Results of individual studies | 20 | For all outcomes considered (benefits or harms), present, for each study: (a) simple summary data for each intervention group (b) effect estimates and confidence intervals, ideally with a forest plot. | 5-6, Figure 2 |
| Synthesis of results | 21 | Present results of each meta-analysis done, including confidence intervals and measures of consistency. | 5-6, Figure 2, Tables 2 and 3 |
| Risk of bias across studies | 22 | Present results of any assessment of risk of bias across studies (see Item 15). | 5-6, Tables 2 and 3, Table S2 and S3, Figures S1-S12 |
| Additional analysis | 23 | Give results of additional analyses, if done (e.g., sensitivity or subgroup analyses, meta-regression [see Item 16]). | 5-6, Tables 2 and 3, Figure 4 |
| **DISCUSSION** |  |  |  |
| Summary of evidence | 24 | Summarize the main findings including the strength of evidence for each main outcome; consider their relevance to key groups (e.g., healthcare providers, users, and policy makers). | 6-9 |
| Limitations | 25 | Discuss limitations at study and outcome level (e.g., risk of bias), and at review-level (e.g., incomplete retrieval of identified research, reporting bias). | 9 |
| Conclusions | 26 | Provide a general interpretation of the results in the context of other evidence, and implications for future research. | 10 |
| **FUNDING** |  |  |  |
| Funding | 27 | Describe sources of funding for the systematic review and other support (e.g., supply of data); role of founders for the systematic review. | 10 |

*From:* Moher D, Liberati A, Tetzlaff J, Altman DG, The PRISMA Group (2009). Preferred Reporting Items for Systematic Reviews and Meta-Analyses: The PRISMA Statement. PLoS Med 6(6): e1000097. doi:10.1371/journal.pmed1000097

For more information, visit: **www.prisma-statement.org**.

**Supplementary material 3** The code in R for this meta-analysis

| Logarithmic conversion (PNL) | rate<-transform [m1, log=log(event/n)];  shapiro.test(rate$log) |
| --- | --- |
| Logit transformation (PLOGIT) | rate<-transform{m1, logit=log[(event/n)/(1-event/n)]};  shapiro.test(rate$logit) |
| Arcsine transformation (PAS) | rate<-transform{m1, arcsin.size=asin[sqrt(event/(n+1))]};  shapiro.test(rate$arcsin) |
| Double-arcsine transformation (PFT) | rate<-transform{m1,darcsin=0.5*[asin(sqrt(event/(n+1)))+asin((sqrt(event+1)/(n+1)))]};  shapiro.test(rate$darcsin) |
| No transformation (PRAW) | rate<-transform[m1, r= event/n];  shapiro.test(rate$r) |

| Forest plots | forest [meta1, xlim=c(-0.2, 0.8)] |
| --- | --- |
| Funnel chart | funnel (meta1) |
| Egger's test | metabias (meta1, method="linreg") |
| The sensitivity analysis | metainf (meta1, pooled = "random") forest (metainf (meta1, pooled = "random"), xlim=c(0, 0.3)) |
| Subgroup analysis | meta1<-metaprop(event, n, study, data=rate, sm="PLN", incr=0.5, allincr=TRUE, addincr=FALSE, title="", byvar= subgroup title, print.byvar=TRUE) |
| Meta-regression analysis | metareg (meta1, ~covariate title) |

**Supplementary material 4** Studies included in the meta-analysis

| Study ID | Sampling time | Income level | Country | Detection methods | Positive samples/  total samples | Quality score | Quality level | |
| --- | --- | --- | --- | --- | --- | --- | --- | --- |
| **Africa** |  |  |  |  |  |  | |  |
| Erume et al. (2016) | 2012.12-2013.7 | Low | Uganda | ELISA | 2/1665 | 5 | | High |
| Kristina et al. (2016) | 2012.12-2013.7 | Low | Uganda | Others | 1/1665 | 3 | | Middle |
| Khan et al. (2019) | 2017.03-2019.07 | Middle | Egypt | ELISA | 36/331 | 4 | | High |
| **America** |  |  |  |  |  |  | |  |
| Stoffregen et al. (2007) | 2002.10-2003.02 | High | USA | Others | 39/80 | 5 | | High |
| Corn et al. (2009) | 2006-2007 | High | USA | Others | 7/170 | 5 | | High |
| Meirelles-Bartoli et al. (2012) | 2006.07 | Middle | Brazil | RBPT | 254/271 | 3 | | Middle |
| Sandfoss et al. (2012) | 2007.09-2010.03 | High | USA | Others | 10/513 | 4 | | High |
| Musser et al. (2013) | 2005.4 | High | USA | Others | 4/40 | 3 | | Middle |
| Christopher et al. (2017) | 2015.02-2015.03 | High | USA | CFT | 1/47 | 4 | | High |
| He et al. (2017) | 2013.03 | High | USA | RBPT | 0/978 | 3 | | Middle |
| Pedersen et al. (2017) | 2015 | High | USA | Others | 49/376 | 4 | | High |
| Lama et al. (2018) | 2015.03-2016.08 | High | USA | PCR | 18/95 | 4 | | High |
| **Asia** |  |  |  |  |  |  | |  |
| He et al. (2000) | ^a^UN | Middle | China | RBPT | 33/340 | 2 | | Middle |
| Pan et al. (2000) | 1992-1999 | Middle | China | RBPT | 3/3155 | 3 | | High |
| Li et al. (2001) | 1998-1999 | Middle | China | TAT | 34/325 | 3 | | High |
| Chen et al. (2001a) | 2000 | Middle | China | RBPT | 3/897 | 3 | | High |
| Chen et al. (2001b) | 1998-1999 | Middle | China | Others | 4/30 | 3 | | High |
| Gao et al. (2001) | 2000 | Middle | China | RBPT | 99/1113 | 4 | | High |
| Lu et al. (2002) | UN | Middle | China | RBPT | 46/30740 | 2 | | Middle |
| Cao et al. (2002) | UN | Middle | China | RBPT | 0/456 | 2 | | Middle |
| Li et al. (2002) | UN | Middle | China | Others | 0/486 | 2 | | Middle |
| Yang et al. (2002a) | 2000 | Middle | China | RBPT/TAT | 16/1732 | 4 | | High |
| Yang et al. (2002b) | 2001 | Middle | China | RBPT/TAT | 1/600 | 3 | | High |
| Ma et al. (2002) | 2001 | Middle | China | RBPT/TAT | 41/849 | 3 | | High |
| Liu (2003) | 2001-2002 | Middle | China | RBPT/TAT | 0/193 | 3 | | High |
| Liu et al. (2003) | 2000-2002 | Middle | China | RBPT | 0/30933 | 4 | | High |
| Cao et al. (2003) | UN | Middle | China | RBPT | 2/98 | 3 | | High |
| Li et al. (2003) | 2001 | Middle | China | TAT | 4/741 | 3 | | High |
| Yang et al. (2003a) | 1999-2001 | Middle | China | TAT | 0/300 | 3 | | High |
| Yang et al. (2003b) | 2002 | Middle | China | RBPT | 0/36 | 3 | | High |
| Wei et al. (2003) | 2002 | Middle | China | Others | 28/788 | 4 | | High |
| He et al. (2004) | 2004 | Middle | China | RBPT | 0/526 | 3 | | High |
| Zhu et al. (2004) | 2000-2002 | Middle | China | RBPT/TAT | 3/1543 | 3 | | High |
| Wang (2004) | 2003 | Middle | China | TAT | 0/120 | 3 | | High |
| Suo et al. (2004) | 2002 | Middle | China | TAT | 0/106 | 3 | | High |
| Jiang et al. (2004) | 2002.07-08 | Middle | China | RBPT | 1/123 | 3 | | High |
| Gao et al. (2004) | 2000-2003 | Middle | China | RBPT | 0/1200 | 4 | | High |
| Xu et al. (2004) | 2000-2003 | Middle | China | Others | 0/2595 | 3 | | High |
| Zhang (2006) | 1989-1995 | Middle | China | RBPT/TAT | 0/2242 | 3 | | High |
| Zhang et al. (2006) | 2005 | Middle | China | RBPT | 0/989 | 3 | | High |
| Miao et al. (2006) | 2003-2005 | Middle | China | RBPT/TAT | 0/32941 | 4 | | High |
| Chen et al. (2006) | 2005-2006 | Middle | China | RBPT | 0/941 | 3 | | High |
| Xu et al. (2007) | 1983-2004 | Middle | China | UN | 0/37329 | 2 | | Middle |
| Wang (2007) | 2004.12.21-2006.12.20 | Middle | China | RBPT | 0/1257 | 3 | | High |
| Zhong et al. (2007) | UN | Middle | China | TAT | 1/141 | 2 | | Middle |
| Zhang et al. (2008) | 2001-2007 | Middle | China | RBPT | 0/10260 | 4 | | High |
| Wen et al. (2008) | 2006.12.11 | Middle | China | RBPT | 0/119 | 3 | | High |
| Xiao (2008) | 2000-2007 | Middle | China | RBPT | 203/11324 | 4 | | High |
| Yuan et al. (2008) | 2006.5-2007.7 | Middle | China | UN | 1/980 | 3 | | High |
| Luo et al. (2009) | 2007 | Middle | China | RBPT/TAT | 3/720 | 3 | | High |
| Chen (2009) | 2006-2008 | Middle | China | RBPT | 0/4083 | 3 | | High |
| Yu (2010) | 1995-2008 | Middle | China | RBPT/TAT | 0/2056 | 3 | | High |
| Cao et al. (2010) | 2005-2008 | Middle | China | RBPT/TAT | 2/803 | 3 | | High |
| Zhou (2011) | 2001-2004 | Middle | China | RBPT/TAT | 24/274 | 3 | | High |
| Zhou et al. (2011) | 2009.2-2010.12 | Middle | China | RBPT | 0/404 | 2 | | Middle |
| Ha et al. (2011) | 2011.04-06 | Middle | China | RBPT | 0/41 | 3 | | High |
| Zhang et al. (2011) | UN | Middle | China | Others | 0/100 | 2 | | Middle |
| Li et al. (2011) | 1996-2010 | Middle | China | RBPT/TAT | 27/25069 | 4 | | High |
| Yang et al. (2011) | UN | Middle | China | RBPT | 14/302 | 2 | | Middle |
| Wang et al. (2011) | 2003-2009 | Middle | China | RBPT/TAT | 0/102 | 4 | | High |
| Xu (2011) | 2004-2009 | Middle | China | RBPT/TAT | 0/3990 | 3 | | High |
| Guo et al. (2011) | 2006-2010 | Middle | China | RBPT/TAT | 0/296 | 3 | | High |
| Huang et al. (2011) | 2006-2010 | Middle | China | RBPT | 38/1734 | 3 | | High |
| Li et al. (2012) | 2009-2011 | Middle | China | RBPT | 1/10123 | 3 | | High |
| Li (2012a) | 1990-2012 | Middle | China | RBPT | 2/3810 | 4 | | High |
| Li (2012b) | UN | Middle | China | RBPT | 0/200 | 2 | | Middle |
| Gong et al. (2012) | 2008-2011 | Middle | China | RBPT | 0/700 | 4 | | High |
| Wang et al. (2012) | 2000 | Middle | China | RBPT | 29/1187 | 3 | | High |
| Wu et al. (2012) | UN | Middle | China | Others | 37/831 | 2 | | Middle |
| Liu et al. (2012) | UN | Middle | China | RBPT | 4/800 | 2 | | Middle |
| Zuo et al. (2013) | 2008-2012 | Middle | China | RBPT | 0/6158 | 2 | | Middle |
| Zhang (2013) | 2011-2013 | Middle | China | RBPT | 28/564 | 3 | | High |
| Wang et al. (2013) | 1980-2011 | Middle | China | RBPT | 300/22148 | 4 | | High |
| Tao et al. (2013) | 2011 | Middle | China | RBPT | 0/200 | 3 | | High |
| He (2014) | 2012-2013 | Middle | China | RBPT | 0/2280 | 4 | | High |
| Liu et al. (2014) | 2013.1 | Middle | China | TAT | 1/1197 | 3 | | High |
| Lu et al. (2014) | 2009-2013 | Middle | China | RBPT/TAT | 0/2773 | 2 | | Middle |
| You et al. (2014) | 2011-2013 | Middle | China | RBPT | 1/2668 | 3 | | High |
| Wang (2014) | 2013 | Middle | China | RBPT/TAT | 1/901 | 3 | | High |
| Qin (2014) | 2013.01-2013.12 | Middle | China | RBPT | 5/100 | 4 | | High |
| Xie et al. (2014) | 2013.01-2014.03 | Middle | China | RBPT | 0/2217 | 3 | | High |
| Song et al. (2015) | 2014 | Middle | China | RBPT/TAT | 37/675 | 3 | | High |
| Ying. (2015) | 2014.04-2014.09 | Middle | China | RBPT/TAT | 0/1150 | 3 | | High |
| Qin et al. (2015) | 2012-2014 | Middle | China | RBPT | 0/548 | 4 | | High |
| Pu et al. (2015) | 2014 | Middle | China | RBPT/TAT | 0/433 | 3 | | High |
| Kang et al. (2015) | 2014-2015 | Middle | China | RBPT | 0/303 | 4 | | High |
| Zhang et al. (2016) | 2012-2013 | Middle | China | RBPT | 2/1131 | 3 | | High |
| Yang et al. (2016) | 2014.04-2015.04 | Middle | China | RBPT | 0/550 | 3 | | High |
| Tao et al. (2016) | 2015 | Middle | China | TAT | 2/1629 | 3 | | High |
| Liu et al. (2017) | 2016.03-2016.05 | Middle | China | ELISA | 10/600 | 4 | | High |
| Huang et al. (2017) | UN | Middle | China | TAT | 0/154 | 3 | | High |
| Jia et al. (2017) | 2012 | Middle | China | RBPT | 19/1639 | 4 | | High |
| Tao et al. (2017) | 2016 | Middle | China | RBPT | 0/1636 | 3 | | High |
| Wei et al. (2017) | 2012 | Middle | China | RBPT | 18/3343 | 3 | | High |
| Xu et al. (2017) | 2015-2016 | Middle | China | RBPT/TAT | 0/1490 | 4 | | High |
| Zhou (2017) | 2016 | Middle | China | RBPT | 0/30 | 4 | | High |
| Bao et al. (2018) | 2015-2017 | Middle | China | RBPT/TAT | 0/4170 | 3 | | High |
| Chen et al. (2018) | 2012-2016 | Middle | China | RBPT/TAT | 7/20221 | 3 | | High |
| Li et al. (2019a) | 2018.08-2018.11 | Middle | China | RBPT | 0/751 | 3 | | High |
| Li et al. (2019b) | UN | Middle | China | RBPT/ELISA | 18/287 | 3 | | High |
| Shome et al. (2019) | UN | Middle | India | Others | 236/575 | 3 | | Middle |
| Lv et al. (2020) | 2016-2018 | Middle | China | RBPT | 0/1470 | 3 | | High |
| **Europe** |  |  |  |  |  |  | |  |
| Cvetnic et al. (2003) | 1996-1998 | Middle | Croatia | Others | 329/2245 | 4 | | High |
| Dahouk et al. (2005) | 1995-1996 | High | Germany | ELISA | 168/763 | 4 | | High |
| Bergagna et al. (2009) | 2001-2007 | High | Italy | Others | 448/2267 | 4 | | High |
| Cvetnić et al. (2009) | 2000-2004 | Middle | Croatia | Others | 864/93531 | 4 | | High |
| Špičić et al. (2010) | 2008 | Middle | Croatia | RBPT | 181/28520 | 3 | | Middle |
| Wu et al. (2011) | 2008.3-2010.1 | High | Switzerland | Others | 121/252 | 4 | | High |
| Grégoire et al. (2012) | 2003-2007 | High | Belgium | ELISA | 641/1168 | 4 | | High |
| Hälli et al. (2012) | 2005-2008 | High | Finland | RBPT | 0/280 | 3 | | Middle |
| Risco et al. (2012) | 2004.10.12-2005.2.28 | High | Spain | ELISA | 121/204 | 4 | | High |
| Cristian et al. (2014) | 2007-2010 | High | Italy | RBPT | 406/1251 | 4 | | High |
| Cristian et al. (2015) | 2009.11.1-2010.1.31 | High | Italy | ELISA | 35/570 | 4 | | High |
| Giulia et al. (2015) | 2009 | High | Italy | RBPT | 25/28 | 3 | | Middle |
| Bertelloni et al. (2018) | 2015.09-2015.12 | High | Italy | Others | 1/1212 | 4 | | High |
| Grantina‑Ievina et al. (2018) | 2015.01-2016.04 | High | Latvia | Others | 235/1044 | 4 | | High |
| Malmsten et al. (2018) | 2013.01-2015.12 | High | Sweden | UN | 0/286 | 4 | | High |
| **Oceania** |  |  |  |  |  |  | |  |
| C Ridoutt et al. (2014) | 2012-2013 | High | Australia | RBPT | 7/238 | 3 | | Middle |
| Pearson et al. (2014) | UN | High | Australia | CFT | 9/83 | 2 | | Middle |

^a^UN: Unclear.

**Table S5** Included studies and their quality scores

| **Study No.** | **Reference ID** | **No. tested** | **No. positive** | **Prevalence** | **Random sampling or not** | **Detection method clearly or not** | **Sampled method detailedly or not** | **Sampled time clearly or not** | **Four or more risk factors or not** | **Score** | **Study Quality** |  |  |  |
| --- | --- | --- | --- | --- | --- | --- | --- | --- | --- | --- | --- | --- | --- | --- |
| 1 | Li et al. (2012) | 10123 | 1 | 0.01% | ^a^N | ^b^Y | N | Y | Y | 3 | high |  |  |  |
| 2 | Li (2012a) | 3810 | 2 | 0.05% | Y | Y | N | Y | Y | 4 | high |  |  |  |
| 3 | Li (2012b) | 200 | 0 | 0.00% | Y | Y | N | N | N | 2 | middle |  |  |  |
| 4 | Gong et al. (2012) | 700 | 0 | 0.00% | Y | Y | N | Y | Y | 4 | high |  |  |  |
| 5 | Zuo et al. (2013) | 6158 | 0 | 0.00% | N | Y | N | Y | N | 2 | middle |  |  |  |
| 6 | Zhang (2013) | 564 | 28 | 4.96% | N | Y | N | Y | Y | 3 | high |  |  |  |
| 7 | Wang et al. (2013) | 22148 | 300 | 1.35% | Y | Y | N | Y | Y | 4 | high |  |  |  |
| 8 | Wang et al. (2012) | 1187 | 29 | 2.44% | N | Y | N | Y | Y | 3 | high |  |  |  |
| 9 | Wu et al. (2012) | 831 | 37 | 4.45% | N | Y | N | N | Y | 2 | middle |  |  |  |
| 10 | Tao et al. (2013) | 200 | 0 | 0.00% | N | Y | N | Y | Y | 3 | high |  |  |  |
| 11 | He (2014) | 2280 | 0 | 0.00% | Y | Y | N | Y | Y | 4 | high |  |  |  |
| 12 | Liu et al. (2014) | 1197 | 1 | 0.08% | N | Y | N | Y | Y | 3 | high |  |  |  |
| 13 | Lu et al (2014) | 2773 | 0 | 0.00% | N | Y | N | Y | N | 2 | middle |  |  |  |
| 14 | You et al (2014) | 2668 | 1 | 0.04% | N | Y | N | Y | Y | 3 | high |  |  |  |
| 15 | Wang (2014) | 901 | 1 | 0.11% | N | Y | N | Y | Y | 3 | high |  |  |  |
| 16 | Qin. (2014) | 100 | 5 | 5.00% | Y | Y | N | Y | Y | 4 | high |  |  |  |
| 17 | Xie et al. (2014) | 2217 | 0 | 0.00% | N | Y | N | Y | Y | 3 | high |  |  |  |
| 18 | Song et al. (2015) | 675 | 37 | 5.48% | N | Y | N | Y | Y | 3 | high |  |  |  |
| 19 | Ying. (2015) | 1150 | 0 | 0.00% | N | Y | N | Y | Y | 3 | high |  |  |  |
| 20 | Qin et al. (2015) | 548 | 0 | 0.00% | Y | Y | N | Y | Y | 4 | high |  |  |  |
| 21 | Zhang et al. (2016) | 1131 | 2 | 0.18% | N | Y | N | Y | Y | 3 | high |  |  |  |
| 22 | Yang et al. (2016) | 550 | 0 | 0.00% | N | Y | N | Y | Y | 3 | high |  |  |  |
| 23 | Pu et al. (2015) | 433 | 0 | 0.00% | N | Y | N | Y | Y | 3 | high |  |  |  |
| 24 | Tao et al. (2016) | 1629 | 2 | 0.12% | N | Y | N | Y | Y | 3 | high |  |  |  |
| 25 | Liu et al. (2017) | 600 | 10 | 1.67% | Y | Y | N | Y | Y | 4 | high |  |  |  |
| 26 | He et al. (2000) | 340 | 33 | 9.71% | N | Y | N | N | Y | 2 | middle |  |  |  |
| 27 | Li et al. (2001) | 325 | 34 | 10.46% | N | Y | N | Y | Y | 3 | high |  |  |  |
| 28 | Pan et al. (2000) | 3155 | 3 | 0.10% | N | Y | N | Y | Y | 3 | high |  |  |  |
| 29 | Chen et al. (2001a) | 897 | 3 | 0.33% | N | Y | N | Y | Y | 3 | high |  |  |  |
| 30 | Chen et al. (2001b) | 30 | 4 | 13.33% | N | Y | N | Y | Y | 3 | high |  |  |  |
| 31 | Gao et al. (2001) | 1113 | 99 | 8.89% | Y | Y | N | Y | Y | 4 | high |  |  |  |
| 32 | Lu et al. (2002) | 30740 | 46 | 0.15% | N | Y | N | N | Y | 2 | middle |  |  |  |
| 33 | Cao et al. (2002) | 456 | 0 | 0.00% | N | Y | N | N | Y | 2 | middle |  |  |  |
| 34 | Li et al. (2002) | 486 | 0 | 0.00% | N | Y | N | N | Y | 2 | middle |  |  |  |
| 35 | Yang et al. (2002a) | 1732 | 16 | 0.92% | Y | Y | N | Y | Y | 4 | high |  |  |  |
| 36 | Yang et al. (2002b) | 600 | 1 | 0.17% | N | Y | N | Y | Y | 3 | high |  |  |  |
| 37 | Ma et al. (2002) | 849 | 41 | 4.83% | N | Y | N | Y | Y | 3 | high |  |  |  |
| 38 | Liu (2003) | 193 | 0 | 0.00% | N | Y | N | Y | Y | 3 | high |  |  |  |
| 39 | Liu et al. (2003) | 30933 | 0 | 0.00% | Y | Y | N | Y | Y | 4 | high |  |  |  |
| 40 | Cao et al. (2003) | 98 | 2 | 2.04% | Y | Y | N | N | Y | 3 | high |  |  |  |
| 41 | Li et al. (2003) | 741 | 4 | 0.54% | N | Y | N | Y | Y | 3 | high |  |  |  |
| 42 | Yang et al. (2003a) | 300 | 0 | 0.00% | N | Y | N | Y | Y | 3 | high |  |  |  |
| 43 | Yang et al. (2003b) | 36 | 0 | 0.00% | N | Y | N | Y | Y | 3 | high |  |  |  |
| 44 | Wei et al. (2003) | 788 | 28 | 3.55% | Y | Y | N | Y | Y | 4 | high |  |  |  |
| 45 | He et al. (2004) | 526 | 0 | 0.00% | N | Y | N | Y | Y | 3 | high |  |  |  |
| 46 | Zhu et al. (2004) | 1543 | 3 | 0.19% | N | Y | N | Y | Y | 3 | high |  |  |  |
| 47 | Wang (2004) | 120 | 0 | 0.00% | N | Y | N | Y | Y | 3 | high |  |  |  |
| 48 | Suo et al. (2004) | 106 | 0 | 0.00% | N | Y | N | Y | Y | 3 | high |  |  |  |
| 49 | Jiang et al. (2004) | 123 | 1 | 0.81% | N | Y | N | Y | Y | 3 | high |  |  |  |
| 50 | Gao et al. (2004) | 600 | 0 | 0.00% | Y | Y | N | Y | Y | 4 | high |  |  |  |
| 51 | Kang et al. (2015) | 303 | 0 | 0.00% | Y | Y | N | Y | Y | 4 | high |  |  |  |
| 52 | Xu et al. (2004) | 2595 | 0 | 0.00% | N | Y | N | Y | Y | 3 | high |  |  |  |
| 53 | Bao et al. (2018) | 4170 | 0 | 0.00% | N | Y | N | Y | Y | 3 | high |  |  |  |
| 54 | Chen et al. (2018) | 20221 | 7 | 0.03% | N | Y | N | Y | Y | 3 | high |  |  |  |
| 55 | Huang et al. (2017) | 154 | 0 | 0.00% | Y | Y | N | N | Y | 3 | high |  |  |  |
| 56 | Jia et al. (2017) | 1639 | 19 | 1.16% | Y | Y | N | Y | Y | 4 | high |  |  |  |
| 57 | Li et al. (2019a) | 751 | 0 | 0.00% | N | Y | N | Y | Y | 3 | high |  |  |  |
| 58 | Li et al. (2019b) | 287 | 18 | 6.27% | Y | Y | N | N | Y | 3 | high |  |  |  |
| 59 | Tao et al. (2017) | 1636 | 0 | 0.00% | N | Y | N | Y | Y | 3 | high |  |  |  |
| 60 | Wei et al. (2017) | 3343 | 18 | 0.54% | N | Y | N | Y | Y | 3 | high |  |  |  |
| 61 | Xu et al. (2017) | 1490 | 0 | 0.00% | Y | Y | N | Y | Y | 4 | high |  |  |  |
| 62 | Zhou (2017) | 30 | 0 | 0.00% | Y | Y | N | Y | Y | 4 | high |  |  |  |
| 63 | lv et al. (2020) | 1470 | 0 | 0.00% | N | Y | N | Y | Y | 3 | high |  |  |  |
| 64 | Zhang (2006) | 2242 | 0 | 0.00% | N | Y | N | Y | Y | 3 | high |  |  |  |
| 65 | Zhang et al. (2006) | 989 | 0 | 0.00% | N | Y | N | Y | Y | 3 | high |  |  |  |
| 66 | Miao et al. (2006) | 32941 | 0 | 0.00% | Y | Y | N | Y | Y | 4 | high |  |  |  |
| 67 | Chen et al. (2006) | 941 | 0 | 0.00% | N | Y | N | Y | Y | 3 | high |  |  |  |
| 68 | Xu et al. (2007) | 37329 | 40 | 0.11% | N | N | N | Y | Y | 2 | middle |  |  |  |
| 69 | Wang (2007) | 1257 | 0 | 0.00% | N | Y | N | Y | Y | 3 | high |  |  |  |
| 70 | Zhong et al. (2007) | 141 | 1 | 0.71% | N | Y | N | N | Y | 2 | middle |  |  |  |
| 71 | Zhang et al. (2008) | 10260 | 0 | 0.00% | Y | Y | N | Y | Y | 4 | high |  |  |  |
| 72 | Wen et al. (2008) | 119 | 0 | 0.00% | N | Y | N | Y | Y | 3 | high |  |  |  |
| 73 | Xiao (2008) | 11324 | 203 | 1.79% | Y | Y | N | Y | Y | 4 | high |  |  |  |
| 74 | Yuan et al. (2008) | 980 | 1 | 0.10% | Y | N | N | Y | Y | 3 | high |  |  |  |
| 75 | Yu (2010) | 2056 | 0 | 0.00% | N | Y | N | Y | Y | 3 | high |  |  |  |
| 76 | Cao et al. (2010) | 803 | 2 | 0.25% | N | Y | N | Y | Y | 3 | high |  |  |  |
| 77 | Luo et al. (2009) | 720 | 3 | 0.42% | N | Y | N | Y | Y | 3 | high |  |  |  |
| 78 | Chen (2009) | 4083 | 0 | 0.00% | N | Y | N | Y | Y | 3 | high |  |  |  |
| 79 | Zhou (2011) | 274 | 24 | 8.76% | N | Y | N | Y | Y | 3 | high |  |  |  |
| 80 | Zhou et al. (2011) | 404 | 0 | 0.00% | N | Y | N | Y | N | 2 | middle |  |  |  |
| 81 | Ha et al. (2011) | 41 | 0 | 0.00% | N | Y | N | Y | Y | 3 | high |  |  |  |
| 82 | Zhang et al. (2011) | 100 | 0 | 0.00% | N | Y | N | N | Y | 2 | middle |  |  |  |
| 83 | Li et al. (2011) | 25069 | 27 | 0.11% | Y | Y | N | Y | Y | 4 | high |  |  |  |
| 84 | Yang et al. (2011) | 302 | 14 | 4.64% | N | Y | N | N | Y | 2 | middle |  |  |  |
| 85 | Wang et al. (2011) | 102 | 0 | 0.00% | Y | Y | N | Y | Y | 4 | high |  |  |  |
| 86 | Xu (2011) | 3990 | 0 | 0.00% | N | Y | N | Y | Y | 3 | high |  |  |  |
| 87 | Guo et al. (2011) | 296 | 0 | 0.00% | N | Y | N | Y | Y | 3 | high |  |  |  |
| 88 | Huang et al. (2011) | 1734 | 38 | 2.19% | N | Y | N | Y | Y | 3 | high |  |  |  |
| 89 | Liu et al. (2012) | 800 | 4 | 0.50% | N | Y | N | N | Y | 2 | middle |  |  |  |
| 90 | He et al. (2017) | 978 | 0 | 0.00% | N | Y | N | Y | Y | 3 | middle |  |  |  |
| 91 | Musser et al (2013) | 40 | 4 | 10.00% | N | Y | N | Y | Y | 3 | middle |  |  |  |
| 92 | Pearson et al. (2014) | 83 | 9 | 10.84% | N | Y | N | N | Y | 2 | middle |  |  |  |
| 93 | Ridoutt,et al (2014) | 238 | 7 | 2.94% | N | Y | N | Y | Y | 3 | middle |  |  |  |
| 94 | Barlozzari et al. (2015) | 28 | 25 | 89.29% | N | Y | N | Y | Y | 3 | middle |  |  |  |
| 95 | Pilo et al. (2015) | 570 | 35 | 6.14% | N | Y | Y | Y | Y | 4 | high |  |  |  |
| 96 | Pilo et al. (2014) | 1251 | 406 | 32.45% | N | Y | Y | Y | Y | 4 | high |  |  |  |
| 97 | Erume et al. (2016) | 1665 | 1 | 0.06% | N | Y | N | Y | Y | 3 | middle |  |  |  |
| 98 | Cleveland et al. (2017) | 47 | 1 | 2.13% | N | Y | Y | Y | Y | 4 | high |  |  |  |
| 99 | Cvetnic et al. (2003) | 2245 | 329 | 14.65% | N | Y | Y | Y | Y | 4 | high |  |  |  |
| 100 | AL Dahouk et al. (2005 | 763 | 168 | 22.02% | N | Y | Y | Y | Y | 4 | high |  |  |  |
| 101 | Bertelloni et al. (2018) | 1212 | 1 | 0.08% | N | Y | Y | Y | Y | 4 | high |  |  |  |
| 102 | Grantina‑Ievina et al. (2018) | 1044 | 235 | 22.51% | N | Y | Y | Y | Y | 4 | high |  |  |  |
| 103 | Khan et al. (2019) | 331 | 36 | 10.88% | N | Y | Y | Y | Y | 4 | high |  |  |  |
| 104 | Lama et al. (2018) | 95 | 18 | 18.95% | N | Y | Y | Y | Y | 4 | high |  |  |  |
| 105 | Malmsten et al. (2018) | 286 | 0 | 0.00% | N | Y | Y | Y | Y | 4 | high |  |  |  |
| 106 | Shome et al. (2019) | 575 | 236 | 41.04% | N | Y | Y | N | Y | 3 | middle |  |  |  |
| 107 | Stoffregen et al. (2007) | 80 | 39 | 48.75% | Y | Y | Y | Y | Y | 5 | high |  |  |  |
| 108 | Corn et al. (2009) | 170 | 7 | 4.12% | Y | Y | Y | Y | Y | 5 | high |  |  |  |
| 109 | Grégoire et al. (2012) | 1168 | 641 | 54.88% | Y | N | Y | Y | Y | 4 | high |  |  |  |
| 110 | Špičić et al. (2010) | 28520 | 181 | 0.63% | N | Y | N | Y | Y | 3 | middle |  |  |  |
| 111 | Hälli et al. (2012) | 280 | 0 | 0.00% | N | Y | N | Y | Y | 3 | middle |  |  |  |
| 112 | Meirelles-Bartoli et al. (2012) | 271 | 254 | 93.73% | N | Y | N | Y | Y | 3 | middle |  |  |  |
| 113 | Sandfoss et al. (2012) | 513 | 10 | 1.95% | N | Y | Y | Y | Y | 4 | high |  |  |  |
| 114 | Cvetnić et al. (2009) | 93531 | 864 | 0.92% | N | Y | Y | Y | Y | 4 | high |  |  |  |
| 115 | Wu et al. (2011) | 252 | 121 | 48.0% | N | Y | Y | Y | Y | 4 | high |  |  |  |
| 116 | Pedersen et al. (2017) | 376 | 49 | 13.0% | N | Y | Y | Y | Y | 4 | high |  |  |  |
| 117 | Bergagna et al. (2009) | 2267 | 448 | 19.8% | N | Y | Y | Y | Y | 4 | high |  |  |  |
| 118 | Erume et al. (2016) | 1665 | 2 | 0.1% | Y | Y | Y | Y | Y | 5 | high |  |  |  |
| 119 | Risco et al. (2012) | 204 | 121 | 59.3% | N | Y | Y | Y | Y | 4 | high |  |  |  |
| ^a^N: No;  ^b^Y: Yes. | | | | | | | | | | | | | | |
|  | | | | | | | | | | | | | | |
| 1. Li, J., Chen, Z. X., Yang, W., Xie, Y.P., Li, C. T., Fu, Q. K., Luo, Y. Q., Li, C. C., Zhao, W. (2012). Surveillance analysis of livestock brucellosis in GuangXi. *Proceedings of the Third Academic Symposium of the Veterinary Public Health Branch of the Chinese Society of Animal Science and Veterinary Medicine*, 476-481. (In Chinese) | | | | | | | | | | | | | |  |
| 1. Li, Q. (2012). Investigation and analysis on Quarantine Monitoring of Brucellosis.*China Cattle Science*, 38, 81-84. (In Chinese) | | | | | | | | | | | | | |  |
| 1. Li, F. (2012). Brief report on surveillance results of brucellosis in Luzhai County.*Guangxi Journal of Animal Husbandry & Veterinary Medicine*, 28, 56. (In Chinese) | | | | | | | | | | | | | |  |
| 1. Gong, G. Y., Zhao, G. Q. (2012). Serological survey of brucellosis of pigs, cattle and sheep in Luxi County.*Yunnan Journal of Animal Science and Veterinary Medicine*, 41, 40-41. (In Chinese) | | | | | | | | | | | | | |  |
| 1. Zuo, S. W., Xue, T., Zhou, Y. H., Yang, Y. L., Yao, Y. B., Lv, R., Wang, S. K. (2013). Results of Brucellosis surveillance in Yuxi City from 2008 to 2012. *China Tropical Medicine*, 13, 1104-1106. (In Chinese) | | | | | | | | | | | | | |  |
| 1. Zhang, S. S. (2013). Investigation report on brucellosis in humans and animals.*Fujian Journal of Animal Husbandry and Veterinary Medicine*, 35, 30-31. (In Chinese) | | | | | | | | | | | | | |  |
| 1. Wang, X. D., Tu, Y. J. (2013). Surveillance and Analysis of Brucellosis of Domestic Animals in Hezhou City.*Guangxi Journal of Animal Husbandry & Veterinary Medicine*, 29, 17-19. (In Chinese) | | | | | | | | | | | | | |  |
| 1. Wang, Q. S., Liu, B. S. (2012). Etiological studies on Reproductive Disorder Syndrome of pigs in Honghe Prefectrue, Yunnan Province. *China Animal Health Inspection*, 29, 42-45. (In Chinese) | | | | | | | | | | | | | |  |
| 1. Wu, X. D., Liu, W. J., Tao, M. J., Li, L., He, H. J., Deng, S. Z., Wang, p. (2012). Survey on Serology of Porcine Brucellosis in Parts of Jiangxi and Fujian Province. *Biological Disaster Science*, 35, 90-92. (In Chinese) | | | | | | | | | | | | | |  |
| 1. Tao, J. S., Xie, C. M. (2013). Luxi County 2011 Monitoring Report on Livestock and Poultry Immunization. Y*unnan Journal of Animal Science and Veterinary Medicine*, 42, 46. (In Chinese) | | | | | | | | | | | | | |  |
| 1. He, Z. L. (2014). The epidemiological investigation of brucellosis in live stocks and its prevention and control, Akesu area.(In Chinese) | | | | | | | | | | | | | |  |
| 1. Liu, D. H., Jiang, H. W., Liang, L., Li, J., Li, Y. R., Li, G. H. (2014). 2013 Shaanxi Imported American Breeding Quarantine. *Animal Husbandry ＆ Veterinary Medicine*, 46, 89-91. (In Chinese) | | | | | | | | | | | | | |  |
| 1. Lu, W. Y., He, F. Y., Li, S. E. (2014). Epidemiological survey of brucellosis in Qingyang City from 2009 to 2013. *Journal of Animal Science and Veterinary Medicine*, 33, 73-74+76. (In Chinese) | | | | | | | | | | | | | |  |
| 1. You, Y. J., Ni, X. W., Wang, J. M., Deng, M., Dong, B. Y., Liu, X. L., Luo, X. l., Du, B. Q. (2014). Surveillance and analysis of brucellosis among animals in Qiannan Prefecture, Guizhou Province. *Animal Husbandry & Veterinary Medicine*, 46, 127-128. (In Chinese) | | | | | | | | | | | | | |  |
| 1. Wang, Y. M. (2014). Serological Investigation of Brucellosis in Large-scale Pig Farms. *Fujian Journal of Animal Husbandry and Veterinary Medicine*, 36, 21-22. (In Chinese) | | | | | | | | | | | | | |  |
| 1. Qin, C. L. (2014). Serological survey of brucellosis in livestock in Rongshui County, *Animals Breeding and Feed,* 13(08), 12-13. doi :10.13300/j.cnki.cn42-1648/s.2014.08.004 (In Chinese) | | | | | | | | | | | | | |  |
| 1. Xie, L. H., Dai, W. G. (2014). Detection and analysis of several important infectious diseases of pigs in Wuzhou City. *Swine Production,* 29(03), 124-125. doi: 10.13257/j.cnki.21-1104/s.2014.03.048 (In Chinese) | | | | | | | | | | | | | |  |
| 1. Song, C. L., Li, Y. N., Li, W. G., Yin, G. F., Li, W. Z. Gao, L. B., Bai, W. B., Shu, X. H. (2015). MPS, PCP,Investigation on infection of TOX and brucellosis. *Heilongjiang Animal Science and Veterinary Medicine,* 6(12), 78-79. doi: 10.13881/j.cnki.hljxmsy.2015.0875 (In Chinese) | | | | | | | | | | | | | |  |
| 1. Ying, Q. X. (2015). Investigation and analysis of the background of major livestock and poultry diseases in Nanping in 2014. *Fujian Journal of Animal Husbandry and Veterinary Medicine,* 37(06), 13-15. (In Chinese) | | | | | | | | | | | | | |  |
| 1. Qin, Y. K., Qin, Q., Lu, X. M., Dai, D. X., Li, Y., Xie, A. H. (2015). Epidemiological survey of brucellosis in Libo County, Guizhou Province. *Guizhou Journal of Animal Husbandry & Veterinary Medicine,* 39(01), 29-30. (In Chinese) | | | | | | | | | | | | | |  |
| 1. Zhang, J. Y., Xu, J., Fang, L. L., Yu, S. M., Sun, G. (2016). Investigation and analysis of epidemic situation of brucellosis in livestock in Heilongjiang province. *Heilongjiang Animal Science and Veterinary Medicine,* 5(10), 104-106. doi: 10.13881/j.cnki.hljxmsy.2016.0873 (In Chinese) | | | | | | | | | | | | | |  |
| 1. Yang, L., Hu, Y. Z., Huang, B. M. (2016). Epidemiological survey of brucellosis in Kaiyang County. *Guizhou Journal of Animal Husbandry & Veterinary Medicine,* 40(01), 40. (In Chinese) | | | | | | | | | | | | | |  |
| 1. Pu, L.Q., Zhang, H., Xiang, Z.L., Wei, J.F., Yang, L.Q., Peng, L.F., Su, Q.S., Zhang, Z.Y. (2015). Surveillance results and countermeasures of livestock brucellosis in Dafang County in 2014. *Contemporary Animal Husbandry, 33*(21), 62-63. (In Chinese) | | | | | | | | | | | | | |  |
| 1. Tao, X., Qiu, L. H., Chen, N., Xie, M. H., Wang, W. C. (2016). Epidemiological survey of animal brucellosis in Chuxiong Prefecture in 2015. *Shanghai Journal of Animal Husbandry and Veterinary Medicine, 61*(03):19. doi: 10.14170/j.cnki.cn31-1278/s.2016.03.007 | | | | | | | | | | | | | |  |
| 1. Liu, P., Li, C., Cheng, Z. L., Chen, M., Chai, Y., Xiao, Y. H., Liu, S. D. (2017). Epidemiological investigation of pig brucellosis and comparison of three detection methods in Jining. *Swine Production, 32*(01):87-88. doi: 10.13257/j.cnki.21-1104/s.2017.01.024 (In Chinese) | | | | | | | | | | | | | |  |
| 1. He, Q. G., Chen, H. C., W, B., Wang, C., Wu, M. Z., Qiu, C. Q., Li, S. C., Lu, Z. X. (2000). Serological investigation of swine chlamydia and brucellosis. *Chinese Veterinary Science, 30*(03), 13-14. doi: 10.16656/j.issn.1673-4696.2000.03.008 | | | | | | | | | | | | | |  |
| 1. Li, D., Li, L., Han, X. L., Chen, X. C. (2001). Diagnosis of SAAT Reaction of Brucellosis and Discussion on Changes of Pig Serum Antibody. *XINJIANGXUMUYE, 17*(03), 28. doi: 10.16795/j.cnki.xjxmy.2001.03.019 | | | | | | | | | | | | | |  |
| 1. Pan, Z. M., Liang, T. K. (2000). Brucellosis surveillance in Huangpu District, Guangzhou. *South China Journal of Preventive Medicine, 41*(01), 66-67. (In Chinese) | | | | | | | | | | | | | |  |
| 1. Chen, Z. M., Tang, H. P., Zhang, C. Y., Zeng, Z. H. (2001). Pig pseudorabies and parvovirus in Zhangzhou City Serological investigation of Japanese encephalitis and brucellosis. *Fujian Journal of Animal Husbandry and Veterinary Medicine, 23*(02), 1. (In Chinese) | | | | | | | | | | | | | |  |
| 1. Chen, Y. H., Li, H., Yang, H. C. (2001). Serological survey of swine reproductive disorders. *Chinese Journal of Veterinary Medicine, 37*(40), 14. (In Chinese) | | | | | | | | | | | | | |  |
| 1. Gao, S. Z., Yang, X. L., Wei, R. S., Han, Q. Y. (2001). Serological survey of brucellosis of swine in large-scale pig farms in Gansu Province. *Chinese Qinghai Journal of Animal and Veterinary Sciences, 31*(05), 22-23. (In Chinese) | | | | | | | | | | | | | |  |
| 1. Lu, J. H., Tan, Z. X., Qiu, L. X., Lu, X. H., Liu, D. X. (2002). monitoring of six infectious diseases that cause swine reproductive syndrome. *Chinese Journal of Preventive Veterinary Medicine, 24*(03), 65-68. (In Chinese) | | | | | | | | | | | | | |  |
| 1. Cao, X. P., Wang, K. R., Zeng, K. X., Li, C. H., Li, X. L., Yang, Y. G., Ma, X. Z. (2002). Survey of epidemic diseases of pigs in Chuxiong Prefecture. *Yunnan Journal of Animal Science and Veterinary Medicine, 31*(03), 11-13. (In Chinese) | | | | | | | | | | | | | |  |
| 1. Li, Y., Zhang, L. A., Yin, T., Chen, L., Huai, Z. J. (2002). Serological survey of epidemic diseases of swine reproductive disorders on large-scale pig farms. *Xinjiang Farm Research of Science and Technology, 25*(04), 19-20. (In Chinese) | | | | | | | | | | | | | |  |
| 1. Yang, B., Sun, G. L., Sun, G., Guo, Z. L., Zhang, J. Y., Tian, S. (2002). Experimental study on monitoring and purification of brucellosis in pig farms in Heilongjiang Province. *Heilongjiang Animal Science and Veterinary Medicine, 45*(03), 38. doi: 10.13881/j.cnki.hljxmsy.2002.03.030 | | | | | | | | | | | | | |  |
| 1. Yang, L., Xu, J. Z., Chen, H., Zhang, T., Xiao, X. Y., Yang, R. S. (2002). Serological Investigation Report on Several Animal Diseases in Qiannan, Guizhou. *Guizhou Journal of Animal Husbandry & Veterinary Medicine, 27*(03), 21. (In Chinese) | | | | | | | | | | | | | |  |
| 1. Ma, H. Q., Wang, X. N., Yao, Y., Xiong, H. Q., Wang, Y. H., Li, P. (2002). Serological survey of brucellosis in livestock in Bijie, Guizhou. *Chinese Journal of Veterinary Medicine, 38*(12), 19-20. (In Chinese) | | | | | | | | | | | | | |  |
| 1. Liu, D.X. (2003). Investigation and Research on the Epidemic Situation of Main Epidemic Diseases on Large-scale Pig Farms in Hunan Province. *Hunan Agricultural University,* 1-43. (In Chinese) | | | | | | | | | | | | | |  |
| 1. Liu, D. X., Tan, Z. X., Qiu, B. G., Qiu, L. X., Lu, L. X., Ren, F. M., Deng, G. Q. (2003). Investigation on the causes of sow reproductive dysfunction syndrome in large-scale pig farms. *China Animal Health Inspection, 22*(12), 31-33. (In Chinese) | | | | | | | | | | | | | |  |
| 1. Cao, X., Peng, D. J. (2003). Serological survey of 5 epidemic diseases including JEV, PR, PPV and other diseases in breeding pigs. *Sichuan Animal & Veterinary Sciences, 30*(S1), 29-32. (In Chinese) | | | | | | | | | | | | | |  |
| 1. Li, X. P., Ma, L.Q., Wang, G. P., Yan, G. F. (2003). Serological investigation of swine brucellosis. *Journal of Domestic Animal Ecology, 24*(04), 37-38. (In Chinese) | | | | | | | | | | | | | |  |
| 1. Yang, Z. R., Wang, H., Zhao, Z. Q., Ban, J. Q., Pu, J. E. (2003). Surveillance report on major swine diseases in Beidao District. *Gansu Animal Husbandry and Veterinary, 34*(01), 18-20. doi: 10.15979/j.cnki.cn62-1064/s.2003.01.009 | | | | | | | | | | | | | |  |
| 1. Yang, X. M., Xie, J., Lin, Y. (2003). Serological diagnosis of swine reproductive disorders. *Sichuan Animal & Veterinary Sciences, 30*(S1), 4-6. (In Chinese) | | | | | | | | | | | | | |  |
| 1. Wei, Y. M., Li, F. (2003). Serological testing of sow reproductive disorders in intensive pig farms. *Animal Husbandry & Veterinary Medicine, 35*(06), 32-34. (In Chinese) | | | | | | | | | | | | | |  |
| 1. He, R. Y., Zhong, Y. S., Xu, H., Peng, G. H. (2004). Surveillance of animal diseases in Zunyi City. *Guizhou Journal of Animal Husbandry & Veterinary Medicine, 28*(06), 22. (In Chinese) | | | | | | | | | | | | | |  |
| 1. Zhu, F. Q., Meng, Z. H., Chen, S. M., Zhang, G. S., Tao, R. X., Cai, R. F. (2004). Investigation on Brucellosis of Domestic Animals in Luliang County. *Yunnan Journal of Animal Science and Veterinary Medicine, 3*3(01), 11-12. (In Chinese) | | | | | | | | | | | | | |  |
| 1. Wang, G. P. (2004). Detection of reproductive disorders of sows in large scale pig farms in Qinghai Province. *Chinese Veterinary Science, 34*(03), 67-68. doi: 10.16656/j.issn.1673-4696.2004.03.020 | | | | | | | | | | | | | |  |
| 1. Suo, N. C., Cui, H. L., Li, Y. L., Zhou, Y. L. (2004). Animal Disease Surveillance Report of Ledu County. *Chinese Qinghai Journal of Animal and Veterinary Sciences, 34*(04), 47. (In Chinese) | | | | | | | | | | | | | |  |
| 1. Jiang, W. S., Luo, Z. Q., Chen, Y. (2004). Epidemiological investigation of epidemic diseases of swine reproductive disorders. *Journal of Animal Science and Veterinary Medicine, 23*(02), 51-53. (In Chinese) | | | | | | | | | | | | | |  |
| 1. Gao, M. Q., Mao, G. C., Xu, Y. Z. (2004). Serological survey of brucellosis in Yuqing County. *China Animal Health Inspection, 21*(02), 29. (In Chinese) | | | | | | | | | | | | | |  |
| 1. Kang, W. B., Song J. G., Liu, Y., Xue, X. J., Zhou, F., Dou, L., Wei, B. X. (2015). Serological Investigation of Vertically Transmitted Epidemic Diseases of Pigs in Yuzhong County. *Chin J Tradit Vet Sci,* 59(08), 8-9. | | | | | | | | | | | | | |  |
| 1. Xu, W. M., Jin, X. Y., Shi, S. F., Yang, Y., Gao, L. L. (2004). Prevention and Surveillance of Brucellosis in Zhejiang Province. *The 7th Epidemiology Academic Exchange Conference in East China,* 248-250. (In Chinese) | | | | | | | | | | | | | |  |
| 1. Bao, T.T., Liao, F., Yang, X.F., Zhao, X.M., Mo, X.H. (2018). Serological survey of animal brucellosis infection in Qiandongnan Prefecture from 2015 to 2017. *Guizhou Journal of Animal Husbandry & Veterinary Medicine*, 42(06), 54-55. (In Chinese) | | | | | | | | | | | | | |  |
| 1. Chen, S. W., Zhou, Z.H., Liu, X. Q., Chen, L. P., Wang, Z.Q., Zhang, X.P., (2018). Investigation on serum antibody of animal brucellosis in Dongguan. *Chinese Journal of Animal Husbandry and Veterinary Medicine,* 34(08),12-14. doi: 10.3969/J.ISSN.1671-6027.2018.08.008 (In Chinese) | | | | | | | | | | | | | |  |
| 1. Huang, S.W., Min, F.G., Wu, R.K., Chen, M.L., Wang, X. L., Pan, J.C. (2017).Microbiological and parasitological investigation of experimental minipigs in Guangdong province. *Chinese Journal of Comparative Medicine,* 27(10), 69-73+79. (In Chinese) | | | | | | | | | | | | | |  |
| 1. Jia, W.Y., Wei, Y.M., Qian, Z.B. (2017). Report on Epidemiological and Serological of Major Diseases in Zhang ye City. *Journal of Animal Science and Veterinary Medicine,* 36(02), 73-75+78. (In Chinese) | | | | | | | | | | | | | |  |
| 1. Li, Y. L., Yang, Y. M., Li, D. F. (2019). Serological survey of brucellosis in livestock. *The Chinese Livestock and Poultry Breeding,* 15(03), 68-69. (In Chinese) | | | | | | | | | | | | | |  |
| 1. Li, Z. Z., Tan, Q. H. (2019). Emergency Epidemiological Investigation on an Outbreak of Swine Brucellosis in Hunan Province. *China Animal Health Inspection,* 36(03), 13-17. doi: 10.3969/j.issn.1005-944X.2019.03.003 (In Chinese) | | | | | | | | | | | | | |  |
| 1. Tao, X., Yang, D. Y., Li, Y. L., Yang,Y. N., Yang, J. (2017). Epidemiological survey of animal brucellosis in Chuxiong Prefecture in 2016, *Shanghai Journal of Animal Husbandry and Veterinary Medicine,* 62(05), 62-63. doi: 10.14170/j.cnki.cn31-1278/s.2017.05.020 (In Chinese) | | | | | | | | | | | | | |  |
| 1. Wei, Y. M., Qian, Z. B. (2017). Report on Epidemiological and Serological of Major Diseases on Scale Swine Farms. *Journal of Animal Science and Veterinary Medicine,* 36(01), 97-101. (In Chinese) | | | | | | | | | | | | | |  |
| 1. Xu, D., Zhu, W. J., Yin, F. F., Zhou, Y., Zhang, H. J. (2017). Surveillance and analysis of brucellosis among animals in Weihai from 2015 to 2016. *Heilongjiang Animal Science and Veterinary Medicine*, 60(24), 115-117. doi: 10.13881/j.cnki.hljxmsy.2017.2082 (In Chinese) | | | | | | | | | | | | | |  |
| 1. Zhou, M. J. (2017). Serological antibody monitoring of common epidemics in a wild boar farm in Fuling District. *Agricultural Development & Equipments*, 23(05), 93. (In Chinese) | | | | | | | | | | | | | |  |
| 1. Lv, Y. Q., Huang, D. F., Yao, X. J., Zhang, Y. C., Wang, Y. W., Kang, X. J., Liu, X., Bai, R. Y., Qiu, H. L., Wang, M. L., Wang, A. L., Ji, M. M., Zhang, Z. D. (2020). Investigation on the infection of brucellosis in pigs. *China Anim Ind, 29*(2), 51. (In Chinese) | | | | | | | | | | | | | |  |
| 1. Zhang, H. L. (2006). Surveillance and control of brucellosis of livestock in Linxia Prefecture. *Chinese Journal of Veterinary Medicine, 42*(12), 30. | | | | | | | | | | | | | |  |
| 1. Zhang, X. P., Li, Y. E., Wen, Q. P., Lai, X. X. (2006). Serological investigation of 5 kinds of pig diseases in Dongguan. *Progress In Veterinary Medicine, 27*(1), 111-112. doi: 10.16437/j.cnki.1007-5038.2006.01.031 (In Chinese) | | | | | | | | | | | | | |  |
| 1. Miao, L. Y., Sao, Y. C., Dong, J. W. (2006). Investigation on epidemic situation of swine brucellosis in Henan Province. *Henan Journal of Animal Husbandry and Veterinary Medicine, 27*(7), 27-28. | | | | | | | | | | | | | |  |
| 1. Chen, J. M., Weng, S. C., Yang, S., Hong, Y. H., Zhao, W., Chen, L. N. (2006). Report on the surveillance of three epidemic diseases in pig farms in Shantou City. *Guangdong Journal of Animal and Veterinary Science, 31*(6), 33-34. | | | | | | | | | | | | | |  |
| 1. Xu, W. M., Shi, S. F., Yang, Y., Jing, X. Y., Wang, H. (2007). Epidemic situation and exploration of the prevention and control strategies on Brucel losis in Zhej iang Province. *Chinese Rural Health Service Administration, 27*(3), 209-211. | | | | | | | | | | | | | |  |
| 1. Wang, D. L. (2007). Research and prevention of swine reproduce disease in Scaled pig stations of haikou city. *Huazhong Agricultural University*. | | | | | | | | | | | | | |  |
| 1. Zhong, H. S., Niu, X. Y. (2007). Serological survey of brucellosis in swine of Qinghai Huzhu. *Chinese Qinghai Journal of Animal and Veterinary Sciences, 37*(4), 25. | | | | | | | | | | | | | |  |
| 1. Zhang, L. X., Zhao, M., Mo, Y. F., Tong, L. (2008). Serological investigation of brucellosis in livestock from 2001 to 2007 in Wenshan Prefecture. *China Animal Health Inspection, 25*(10), 36. | | | | | | | | | | | | | |  |
| 1. Wen, F. Y., Wang, G. L., Yu, G. F. (2008). Serological survey and analysis of major swine diseases in Miyun County, Beijing in 2006. *Beijing Agriculture, 28*(24), 4-6. | | | | | | | | | | | | | |  |
| 1. Xiao, G. P. (2008). Serological survey of brucellosis in Qujing City. *China Animal Health Inspection, 25*(11), 37. | | | | | | | | | | | | | |  |
| 1. Yuan, L. G., Pu, J. W., Long, W. Z., Miao, W. G., Sha, L., Tuo, E. G., Lu, J. W., Li, C. (2008). Analysis and countermeasures of pig disease monitoring in breeding area. *Animal Science Abroad (Pigs and Poultry), 26*(2), 85-86. | | | | | | | | | | | | | |  |
| 1. Yu, H. Y. (2010). Analysis of surveillance and purification of brucellosis among animals in Ganzhou. *China Animal Health Inspection, 27*(7), 53-54. | | | | | | | | | | | | | |  |
| 1. Cao, X. P., Zhang, Y. F., Gao, Z. S., Zhang, L. F., Rao, M., Li, G. F. (2010). Seroepidemiological study on the main infectious diseases of swine reproductive disorders. *China Animal Health Inspection, 27*(3), 49-52. | | | | | | | | | | | | | |  |
| 1. Luo, M. X., Zhou, B. J., Wang, K. G., Wen, M., Wang, D. S., Han, J. B., Yang, J. (2009). Serological Detection for Brucellosis of Livestock in Guizhou Province. *Guizhou Agricultural Sciences, 37*(1), 109-110. | | | | | | | | | | | | | |  |
| 1. Chen, C. L. (2009). Detection and epidemic characteristics of major epidemic diseases in large-scale pig farms. *Journal of Animal Science and Veterinary Medicine, 28*(6), 35-36. | | | | | | | | | | | | | |  |
| 1. Zhou, Q. Y. (2011). Epidemiological survey of swine reproductive disorder syndrome in Mengzi County, Yunnan Province. *Animals Breeding and Feed, 10*(2), 7-10. doi: 10.13300/j.cnki.cn42-1648/s.2011.02.005 (In Chinese) | | | | | | | | | | | | | |  |
| 1. Zhou, J. Z., Gong, X. W., Cao, X. A., Zheng, F. Y., Lin, G. Z., Wang, G. H., Fei, Y. Y., Qiu, C. Q. (2011). Investigation on the epidemic situation of animal brucellosis in some provinces (regions) in China from 2009 to 2010. *Proceedings of the 3rd National Conference on Zoonoses,* 2011.09.01. | | | | | | | | | | | | | |  |
| 1. Ha, X. B. T., Xin, N., Wu, N. Q. M., Dao, E. J. (2011). Investigation on Brucellosis of Domestic Animals in Hezhou County, Bazhou. *Xinjiang Animal Husbandry, 27*(11), 32-33. doi: 10.16795/j.cnki.xjxmy.2011.11.008 (In Chinese) | | | | | | | | | | | | | |  |
| 1. Zhang, J. N., Song, C. X., Gu, W. W., Wu, Q. H. (2011). Serological survey of infectious diseases of 10 mini-pigs in Tibet. *Chinese Journal of Comparative Medicine, 21*(5), 74-76. | | | | | | | | | | | | | |  |
| 1. Li, C. T., Jiang, Y. B., Hua, M. Z., Yan, M., Huang, H. L., Chen, J. F., Chen, Z. X. (2011). Serological monitoring of swine brucellosis in Long'an County and its control strategy. *Guangxi Journal of Animal Husbandry & Veterinary Medicine, 27*(5), 285-287. | | | | | | | | | | | | | |  |
| 1. Yang, Y., Ta, Y. F., Yang, H. H., Niu, M. C., Yang,G. S. (2011). Serological Survey on Porcine Brucellosis in Yunnan Province. *Modern Agricultural Science and Technology, 40*(4), 319+324. | | | | | | | | | | | | | |  |
| 1. Wang, Y., Chen, G. C., Liu, Z. B., Gong, X. J., Huang, H. W. (2011). Investigation on the prevalence of brucellosis in some large hydropower projects in Guizhou Province. *Journal of Medical Pest Control, 27*(9), 850. doi: 10.3969/j.issn.1003-6245.2011.09.028 (In Chinese) | | | | | | | | | | | | | |  |
| 1. Xu, Y. P. (2011). Analysis of Surveillance Results of Brucellosis in Domestic Animals in Gansu Province. *Animal Husbandry & Veterinary Medicine, 43*(6), 112. | | | | | | | | | | | | | |  |
| 1. Guo, G. R., Luo, Y. F., Luo, M., Xiao, M. C., Hu, M. X., Pan, S. H. (2011). Serological survey of brucellosis in livestock in Longli County, Guizhou. *Shanghai Journal of Animal Husbandry and Veterinary Medicine, 56*(5), 42-43. | | | | | | | | | | | | | |  |
| 1. Huang, S. M., Yang, Z. S., Shi, Y. L., Zeng, Z. Y., Qi, T. T. (2011). Survey of animal brucellosis in Haikou area. *China Tropical Medicine*, *11*(10), 1240-1241. doi: 10.13604/j.cnki.46-1064/r.2011.10.004 (In Chinese) | | | | | | | | | | | | | |  |
| 1. Liu, J., Tang, D. Y., Zeng, Z. Y., Luo, X. F., Li, C. Y., Gan, Z. L., Wang, F., Hao, F. (2012). Serological investigation of swine brucellosis in a large-scale pig far. *Swine Industry Science, 29*(10), 49-50. | | | | | | | | | | | | | |  |
| 1. He, C. W., Jiang, Q. X., Wei, J. Y., Liao, C. L. Chen, L. J., Pan, B. J., Liao, H. Y. (2017). Report on inspection and quarantine of imported US boars. *Guangxi Journal of Animal Husbandry & Veterinary Medicine, 33*(01):48-49. (In Chinese) | | | | | | | | | | | | | |  |
| 1. Musser, J. M. B., Schwartz, A. L., Srinath, I., Waldrup, K. A. (2013). Use of serology and bacterial culture to determine prevalence of Brucella spp. In feral Swine (sus scrofa) in proximity to a beef cattle herd positive for Brucella suis and Brucella. *J Wildl Dis*, *49*(2). doi: 10.7589/2012-06-169 | | | | | | | | | | | | | |  |
| 1. Pearson, H. E., Toribio, J. L. M. L., Hernandez-Jover, M., Marshall, D., Lapidge, S. J. (2014). Pathogen presence in feral pigs and their movement around two commercial piggeries in Queensland, Australia. *Vet Rec*, *174*(13). doi: 10.1136/vr.102019 | | | | | | | | | | | | | |  |
| 1. Ridoutt, C., Lee, A., Moloney, B., Massey, P., Charman, N., Jordan, D. (2014). Detection of brucellosis and leptospirosis in feral pigs in New South Wales. *Aust Vet J*, *92*(9). doi: 10.1111/avj.12203 | | | | | | | | | | | | | |  |
| 1. Barlozzari, G., Franco, A., Macrì, G., Lorenzetti, S., Maggiori, F., Dottarelli, S., Maurelli, M., Di Giannatale, E., Tittarelli, M., Battisti, A., Gamberale, F. (2015). First report of Brucella suis biovar 2 in a semi free-range pig farm, Italy. *Vet Ital, 51*(2), 151-154. doi: 10.12834/VetIt.50.3384.1 | | | | | | | | | | | | | |  |
| 1. Pilo, C., Addis, G., Deidda, M., Tedde, M. M., Liciardi, M. (2015). A Serosurvey for Brucellosis in Wild Boar (Sus scrofa) in Sardinia, Italy. *J Wildl Dis,* 51(4), 885–888. doi: 10.7589/2014-11-264 | | | | | | | | | | | | | |  |
| 1. Pilo, C., Tedde, M. T., Orrù, G., Addis, G., Liciardi, M. (2014). Brucella suis infection in domestic pigs in Sardinia (Italy). *Epidemiol. Infect,* 2014, 1-8. doi: 10.1017/S0950268814003513 | | | | | | | | | | | | | |  |
| 1. Erume, J., Roesel, K., Dione, M. M., Ejobi, F., Mboowa, G., Kungu, J. M., Akol, J., Pezo, D., El-Adawy, H., Melzer, F., Elschner, M., Neubauer, H., Grace, D. (2016). Serological and molecular investigation for brucellosis in swine in selected districts of Uganda. *Tropical Animal Health and Production, 48*(6), 1147-55. doi: 10.1007/s11250-016-1067-9 | | | | | | | | | | | | | |  |
| 1. Cleveland, C. A., DeNicola, A., Dubey, J. P., Hill, D. E., Berghaus, R. D., Yabsley, M. J. (2017). Survey for selected pathogens in wild pigs (Sus scrofa) from Guam, Marianna Islands, USA. *Veterinary Microbiology, 205*, 22–25. doi: 10.1016/j.vetmic.2017.05.001 | | | | | | | | | | | | | |  |
| 1. Cvetnic, Z., Mitak, M., Ocepek, M., Lojkic, M., Terzic, S., Jemersic, L., Humski, A., Habrun, B., Sostaric, B., Brstilo, M., Krt, B., Garin-Bastuji, B. (2003). Wild boars (Sus Scrofa) as reservoirs of Brucella suis biovar 2 in Croatia. *Acta Vet Hung, 51*(4), 465-473. doi: 10.1556/AVet.51.2003.4.4 | | | | | | | | | | | | | |  |
| 1. AL Dahouk, S., Nockler, K., Tomaso, H., Splettstoesser, W. D., Jungersen, G., Riber, U., Petry, T., Hoffmann, D., Scholz, H. C., Hensel, A., Neubauer, H. (2005). Seroprevalence of brucellosis, tularemia, and yersiniosis in wild boars (Sus scrofa) from north-eastern Germany. *J Vet Med B Infect Dis Vet Public Health, 52*(10), 444-455. doi: 10.1111/j.1439-0450.2005.00898.x | | | | | | | | | | | | | |  |
| 1. Bertelloni, F., Forzan, M., Turchi, B., Sagona, S., Mazzei, M., Felicioli, A., Fratini, F., Cerri, D. (2018). A Serological Survey on Swine Brucellosis Using Standard Procedures, Dot Blot, and Western Blot in Finisher Pigs in Central-North Italy. *Vet sci*, *5*(4), 86 doi: 10.3390/vetsci5040086 | | | | | | | | | | | | | |  |
| 1. Grantina-Ievina, L., Avsejenko, J., Cvetkova, S., Krastina, D., Streikisa, M., Steingolde, Z., Vevere, I., Rodze, I. (2018). Seroprevalence of Brucella suis in eastern Latvian wild boars (Sus scrofa). *Acta vet Scand*, *60*(1), 19. doi: 10.1186/s13028-018-0373-9 | | | | | | | | | | | | | |  |
| 1. Khan, A.U., Melzer, F., El-Soally, S.A.G.E., Elschner, M.C., Mohamed, S.A., Sayed, A.M.A., Roesler, U., Neubauer, H., El-Adawy, H. (2019). Serological and Molecular Identification of Brucella spp. in Pigs from Cairo and Giza Governorates, Egypt. *Pathogens,* *8*(4), 248 doi: 10.3390/pathogens8040248 | | | | | | | | | | | | | |  |
| 1. Lama, J.K., Bachoon, D.S. (2018). Detection of Brucella suis, Campylobacter jejuni, and Escherichia coli Strains in Feral Pig (Sus scrofa) Communities of Georgia. *Vector borne zoonotic dis,* *18*(7), 350-355 doi: 10.1089/vbz.2017.2187 | | | | | | | | | | | | | |  |
| 1. Malmsten, A., Magnusson, U., Ruiz-Fons, F., González-Barrio, D., Dalin, A. (2018). A serologic survey of pathogens in wild boar (sus scrofa) in swweden. *J wildl dis,* *54*(2), 229-237. doi: 10.7589/2017-05-120 | | | | | | | | | | | | | |  |
| 1. Shome, R., Kalleshamurthy, T., Natesan, K., Jayaprakash, K. R., Byrareddy, K., Mohandoss, N., Sahay, S., Shome, B. R., Hiremath, J., Rahman, H., Barbuddhe, S. B. (2019). Serological and molecular analysis for brucellosis in selected swine herds from Southern India. *J Infect Public Health, 12*(2), 247-251. doi: 10.1016/j.jiph.2018.10.013 | | | | | | | | | | | | | |  |
| 1. Stoffregen, W. C., Olsen, S. C., Jack Wheeler, C., Bricker, B. J., Palmer, M. V., Jensen, A. E., Halling, S. M., Alt, D. P. (2007). Diagnostic characterization of a feral swine herd enzootically infected with Brucella. *J Vet Diagn Invest, 19*(3), 227-37. doi: 10.1177/104063870701900301 | | | | | | | | | | | | | |  |
| 1. Corn, J. L., Cumbee, J. C., Barfoot, R., Erickson, G. A. (2009). Pathogen exposure in feral swine populations geographically associated with high densities of transitional swine premises and commercial swine production. *J Wildl Dis, 45*(3), 713-21. doi: 10.7589/0090-3558-45.3.713 | | | | | | | | | | | | | |  |
| 1. Grégoire, F., Mousset, B., Hanrez, D., Michaux, C., Walravens, K., Linden, A. (2012). A serological and bacteriological survey of brucellosis in wild boar (Sus scrofa) in Belgium. *BMC Vet Res, 8*, 80. doi: 10.1186/1746-6148-8-80 | | | | | | | | | | | | | |  |
| 1. Špičić, S., Zdelar-Tuk, M., Račić, I., Duvnjak, S., Cvetnić, Z. (2010). Serological, bacteriological, and molecular diagnosis of brucellosis in domestic animals in Croatia. *Croat Med J, 51*(4), 320-6. doi: 10.3325/cmj.2010.51.320 | | | | | | | | | | | | | |  |
| 1. Hälli, O., Ala-Kurikka, E., Nokireki, T., Skrzypczak, T., Raunio-Saarnisto, M., Peltoniemi, O. A. T., Heinonen, M. (2012). Prevalence of and risk factors associated with viral and bacterial pathogens in farmed European wild boar. *Vet J, 194*(1), 98-101. doi: 10.1016/j.tvjl.2012.03.008 | | | | | | | | | | | | | |  |
| 1. Meirelles-Bartoli, R. B., Mathias, L. A., Samartino, L. E. (2012). Brucellosis due to Brucella suis in a swine herd associated with a human clinical case in the State of São Paulo, Brazil. *Trop Anim Health Prod, 44*(7), 1575-1579. doi: 10.1007/s11250-012-0108-2 | | | | | | | | | | | | | |  |
| 1. Sandfoss, M. R., DePerno, C. S., Betsill, C. W., Palamar, M. B., Erickson, G., Kennedy-Stoskopf, S. (2012). A Serosurvey for Brucella suis, Classical Swine Fever Virus, Porcine Circovirus Type 2, and Pseudorabies Virus in Feral Swine (Sus scrofa) of Eastern North Carolina. *J Wildl Dis*, 48(2), 462-466. doi: 10.7589/0090-3558-48.2.462 | | | | | | | | | | | | | |  |
| 1. Cvetnić, Ž., Špičić, S., Tončić, J., Majnarić, D., Benić, M., Albert, D., Thiébaud, M., Garin-Bastuji, B. (2009). Brucella suis infection in domestic pigs and wild boar in Croatia. *Rev sci tech, 28*(3), 1057-1067. doi: 10.20506/rst.28.3.1947 2. Wu, N., Abril, C., Hinić, V., Brodard, I., Thür, B., Fattebert, J., Hüssy, D., Ryser-degiorgis, M. (2011). Free-ranging wild boar: a disease threat to domestic pigs in Switzerland?, *J Wildl Dis*, *47*(4), 868-879. doi: 10.7589/0090-3558-47.4.868. 3. Pedersen, K., Bauer, N. E., Olsen, S., Arenas-Gamboa, A. M., Henry, A. C., Sibley, T. D., Gidlewski, T. (2017). Identification of Brucellaspp. in feral swine (Sus scrofa) at abattoirs in Texas, USA, *Zoonoses Public Hlth, 11*(64), 647–654. doi: 0.1111/zph.12359. 4. Bergagna, S., Zoppi, S., Ferroglio, E., Gobetto, M., Dondo, A., Di Giannatale, E., Gennero, M. S., Grattarola, C. (2009). Epidemiologic survey for Brucella suis Biovar 2 in a Wild Boar (Sus scrofa) Population in Northwest Italy, *J Wildl Dis*, *45*(4), 1178-81. doi: 10.7589/0090-3558-45.4.1178. 5. Erume, J., Roesel, K., Dione, M. M., Ejobi, F., Mboowa, G., Kungu, J. M., Akol, J., Pezo, D., El-Adawy, H., Melzer, F., Elschner, M., Neubauer, H., Grace, D. (2016). Serological and molecular investigation for brucellosis in swine in selected districts of Uganda. *Trop Anim Health Prod, 48*(6):1147-55. doi: 10.1007/s11250-016-1067-9. 6. Risco, D., García, A., Serrano, E., Fernandez-Llario, P., Benítez, J. M., Martínez, R., García, W. L., Mendoza, J. H. (2014). High-density dependence but low impact on selected reproduction parameters of Brucella suis biovar 2 in wild boar hunting estates from South-Western Spain. *Transbound Emerg Dis, 61*(6):555-62. doi: 10.1111/tbed.12060. | | | | | | | | | | | | | |  |

**Supplementary material 6** Table of Egger’s test for publication bias

| slope | bias | se. bias | t | df | p-value |  |
| --- | --- | --- | --- | --- | --- | --- |
| 0.040 | 4.885 | 1.393 | 3.507 | 117 | 0.001 |  |

**Supplementary material 7** Figure of Egger’s test for publication bias

0

100

200

300

400

500

600

0

10

20

30

40

50

60

Inverse of standard error

Standardised treatment effect (z-score)

**Supplementary material 8** Funnel plot with a trim and fill analysis for the publication bias test

-1.0

-0.5

0.0

0.5

1.0

0.08

0.06

0.04

0.02

0.00

Freeman-Tukey Double Arcsine Transformed Proportion

Standard Error

**Supplementary material 9** Funnel plot with pseudo 95% confidence intervals for the examination of publication bias by continent

0.0

0.5

1.0

0.08

0.06

0.04

0.02

0.00

Freeman-Tukey Double Arcsine Transformed Proportion

Standard Error

**Supplementary material 10** Funnel plot with pseudo 95% confidence intervals for the examination of publication bias by sampling years

0.0

0.5

1.0

0.10

0.08

0.06

0.04

0.02

0.00

Freeman-Tukey Double Arcsine Transformed Proportion

Standard Error

**Supplementary material 11** Funnel plot with pseudo 95% confidence intervals for the examination of publication bias by income level

0.0

0.5

1.0

0.08

0.06

0.04

0.02

0.00

Freeman-Tukey Double Arcsine Transformed Proportion

Standard Error

**Supplementary material 12** Funnel plot with pseudo 95% confidence intervals for the examination of publication bias by detection method

0.0

0.5

1.0

0.15

0.10

0.05

0.00

Freeman-Tukey Double Arcsine Transformed Proportion

Standard Error

**Supplementary material 13** Funnel plot with pseudo 95% confidence intervals for the examination of publication bias by season

0.0

0.2

0.4

0.6

0.8

1.0

1.2

1.4

0.08

0.06

0.04

0.02

0.00

Freeman-Tukey Double Arcsine Transformed Proportion

Standard Error

**Supplementary material 14** Funnel plot with pseudo 95% confidence intervals for the examination of publication bias by gender

0.0

0.5

1.0

0.15

0.10

0.05

0.00

Freeman-Tukey Double Arcsine Transformed Proportion

Standard Error

**Supplementary material 15** Funnel plot with pseudo 95% confidence intervals for the examination of publication bias by age

-0.4

-0.2

0.0

0.2

0.4

0.6

0.20

0.15

0.10

0.05

0.00

Freeman-Tukey Double Arcsine Transformed Proportion

Standard Error

**Supplementary material 16** Funnel plot with pseudo 95% confidence intervals for the examination of publication bias by feeding mode

0.0

0.2

0.4

0.6

0.8

1.0

1.2

0.08

0.06

0.04

0.02

0.00

Freeman-Tukey Double Arcsine Transformed Proportion

Standard Error

**Supplementary material 17** Funnel plot with pseudo 95% confidence intervals for the examination of publication bias by pig classification

-0.2

0.0

0.2

0.4

0.6

0.8

0.15

0.10

0.05

0.00

Freeman-Tukey Double Arcsine Transformed Proportion

Standard Error

**Supplementary material 18** Funnel plot with pseudo 95% confidence intervals for the examination of publication bias by study quality

0.0

0.5

1.0

0.08

0.06

0.04

0.02

0.00

Freeman-Tukey Double Arcsine Transformed Proportion

Standard Error

**Supplementary material 19** Results of the sensitivity analysis. After omitting one study at a time, the random effects model was used to reanalyze the remaining studies to verify the impact of one study on the overall results.


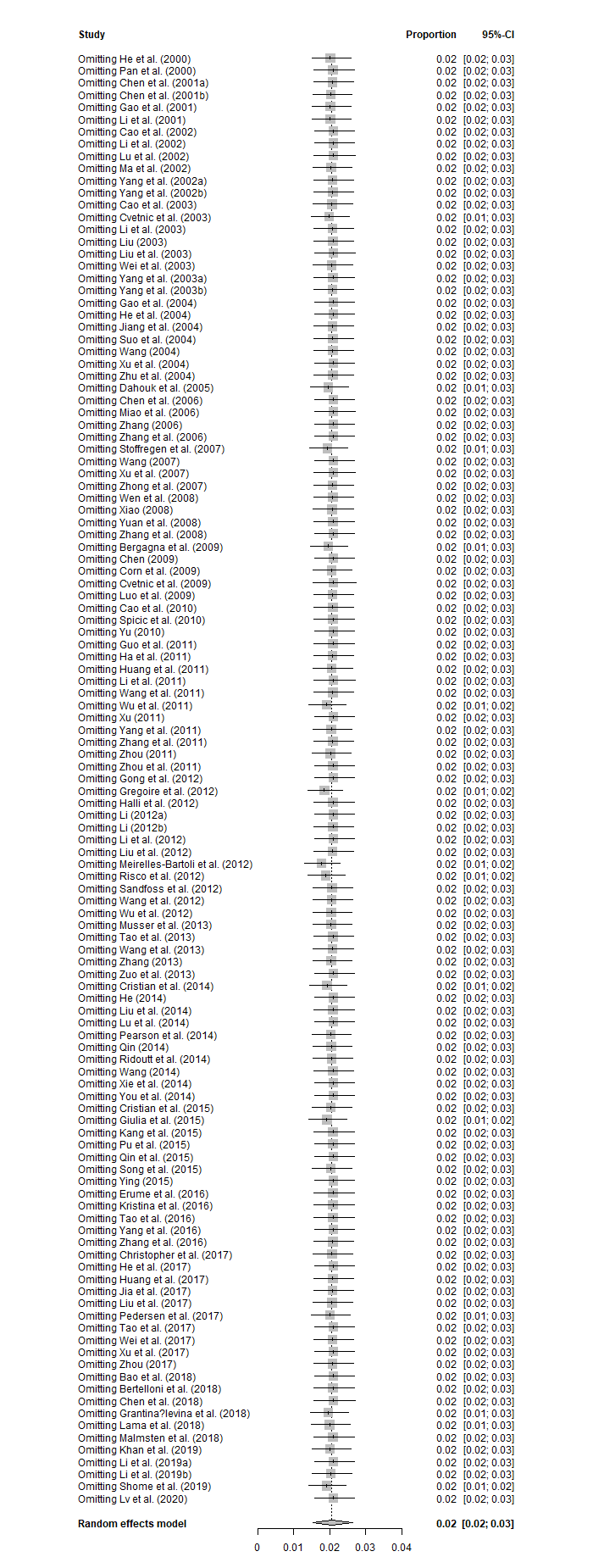

Supplement: Supplementary file 1 [file Data_Sheet_1.docx]
